# Supplementary material for: Implications of the Niche Partitioning and Coexistence of Two Resident Parasitoids for Drosophila suzukii Management in Non-Crop Areas
Source: Insects. 2023 Feb 23;14(3):222. doi: 10.3390/insects14030222 (PMC10051101; doi:10.3390/insects14030222)
Supplement: Supplementary file 1 [file insects-14-00222-s001.zip › File S1 Raw data_Buonocore Biancheri et al._full data.pdf]

The column names in the Raw Data File were modified to be self-explanatory, and may differ from the names used in the statistical analyses

Collecting Date	Habitat	Fly Species	N° of Drosophilid puparia	*Trichopria anastrephae* parasitism	*Pachycrepoideus vindemiae* parasitism
5/12/2016	Inside	Dsuz	2	1	0
5/12/2016	Inside	Dsuz	3	1	0
5/12/2016	Inside	Dsuz	6	2	0
5/12/2016	Inside	Dsuz	4	2	0
5/12/2016	Inside	Dsuz	2	0	0
5/12/2016	Inside	Dsuz	1	0	0
5/12/2016	Inside	Dsuz	2	0	0
5/12/2016	Inside	Dsuz	5	1	0
5/12/2016	Inside	Dsuz	9	3	1
5/12/2016	Inside	Dsuz	4	2	1
5/12/2016	Inside	Dsuz	3	1	0
5/12/2016	Inside	Dsuz	5	1	0
5/12/2016	Inside	Dsuz	9	3	0
5/12/2016	Inside	Dsuz	1	0	0
5/12/2016	Inside	Dsuz	4	1	0
5/12/2016	Inside	Dsuz	3	1	0
5/12/2016	Inside	Dsuz	2	0	0
5/12/2016	Inside	Dsuz	5	2	0
5/12/2016	Inside	Dsuz	2	0	0
5/12/2016	Inside	Dsuz	1	0	0
15/12/2016	Inside	Dsuz	4	2	0
15/12/2016	Inside	Dsuz	2	1	0
15/12/2016	Inside	Dsuz	7	2	2
15/12/2016	Inside	Dsuz	9	2	2
15/12/2016	Inside	Dsuz	6	2	2
15/12/2016	Inside	Dsuz	1	1	0
15/12/2016	Inside	Dsuz	3	1	0
15/12/2016	Inside	Dsuz	7	2	1
15/12/2016	Inside	Dsuz	6	2	0
15/12/2016	Inside	Dsuz	9	2	1
15/12/2016	Inside	Dsuz	3	1	0
15/12/2016	Inside	Dsuz	2	1	0
15/12/2016	Inside	Dsuz	6	2	1
15/12/2016	Inside	Dsuz	3	1	0
15/12/2016	Inside	Dsuz	6	3	0
15/12/2016	Inside	Dsuz	8	3	1
15/12/2016	Inside	Dsuz	3	1	0
15/12/2016	Inside	Dsuz	6	3	0
15/12/2016	Inside	Dsuz	7	3	1
15/12/2016	Inside	Dsuz	4	2	0
26/12/2016	Inside	Dsuz	2	1	0

Codes	
Inside =	drosophilid puparia inside fruit
Outside =	drosophilid puparia outside fruit
Soil =	drosophilid puparia buried
Dsuz =	*Drosophila suzukii*
Dspp =	saprophytic drosophilid especies

26/12/2016	Inside	Dsuz	9	2	0
26/12/2016	Inside	Dsuz	6	2	1
26/12/2016	Inside	Dsuz	9	2	0
26/12/2016	Inside	Dsuz	3	1	0
26/12/2016	Inside	Dsuz	3	1	0
26/12/2016	Inside	Dsuz	5	1	0
26/12/2016	Inside	Dsuz	3	1	0
26/12/2016	Inside	Dsuz	6	1	1
26/12/2016	Inside	Dsuz	9	2	1
26/12/2016	Inside	Dsuz	6	1	0
26/12/2016	Inside	Dsuz	7	1	0
26/12/2016	Inside	Dsuz	6	1	0
26/12/2016	Inside	Dsuz	10	3	2
26/12/2016	Inside	Dsuz	5	1	0
26/12/2016	Inside	Dsuz	4	1	0
26/12/2016	Inside	Dsuz	2	1	0
26/12/2016	Inside	Dsuz	8	2	1
26/12/2016	Inside	Dsuz	3	1	0
26/12/2016	Inside	Dsuz	5	1	0
5/1/2017	Inside	Dsuz	4	0	0
5/1/2017	Inside	Dsuz	3	0	0
5/1/2017	Inside	Dsuz	7	2	0
5/1/2017	Inside	Dsuz	4	2	0
5/1/2017	Inside	Dsuz	10	3	0
5/1/2017	Inside	Dsuz	2	0	0
5/1/2017	Inside	Dsuz	4	2	0
5/1/2017	Inside	Dsuz	6	2	0
5/1/2017	Inside	Dsuz	3	0	0
5/1/2017	Inside	Dsuz	2	0	0
5/1/2017	Inside	Dsuz	3	1	0
5/1/2017	Inside	Dsuz	2	0	0
5/1/2017	Inside	Dsuz	4	1	0
5/1/2017	Inside	Dsuz	2	1	0
5/1/2017	Inside	Dsuz	1	1	0
5/1/2017	Inside	Dsuz	5	1	1
5/1/2017	Inside	Dsuz	2	1	0
5/1/2017	Inside	Dsuz	2	1	0
5/1/2017	Inside	Dsuz	1	0	0
5/1/2017	Inside	Dsuz	2	1	0
16/1/2017	Inside	Dsuz	4	1	0
16/1/2017	Inside	Dsuz	6	2	0
16/1/2017	Inside	Dsuz	3	1	0
16/1/2017	Inside	Dsuz	4	1	0
16/1/2017	Inside	Dsuz	9	2	1
16/1/2017	Inside	Dsuz	2	1	0
16/1/2017	Inside	Dsuz	3	1	0
16/1/2017	Inside	Dsuz	6	2	0

16/1/2017	Inside	Dsuz	4	1	1
16/1/2017	Inside	Dsuz	3	1	0
16/1/2017	Inside	Dsuz	5	2	0
16/1/2017	Inside	Dsuz	2	1	0
16/1/2017	Inside	Dsuz	3	1	0
16/1/2017	Inside	Dsuz	2	1	0
16/1/2017	Inside	Dsuz	4	1	1
16/1/2017	Inside	Dsuz	2	1	0
16/1/2017	Inside	Dsuz	3	1	0
16/1/2017	Inside	Dsuz	2	1	0
16/1/2017	Inside	Dsuz	2	1	0
16/1/2017	Inside	Dsuz	4	1	0
26/1/2017	Inside	Dsuz	2	0	0
26/1/2017	Inside	Dsuz	4	2	0
26/1/2017	Inside	Dsuz	3	1	0
26/1/2017	Inside	Dsuz	3	1	0
26/1/2017	Inside	Dsuz	0	0	0
26/1/2017	Inside	Dsuz	3	1	0
26/1/2017	Inside	Dsuz	3	1	0
26/1/2017	Inside	Dsuz	0	0	0
26/1/2017	Inside	Dsuz	4	1	0
26/1/2017	Inside	Dsuz	3	1	0
26/1/2017	Inside	Dsuz	3	2	0
26/1/2017	Inside	Dsuz	3	2	1
26/1/2017	Inside	Dsuz	5	1	0
26/1/2017	Inside	Dsuz	6	2	0
26/1/2017	Inside	Dsuz	4	1	0
26/1/2017	Inside	Dsuz	3	1	0
26/1/2017	Inside	Dsuz	4	1	0
26/1/2017	Inside	Dsuz	6	1	1
26/1/2017	Inside	Dsuz	3	1	0
26/1/2017	Inside	Dsuz	4	1	0
5/12/2016	Outside	Dsuz	4	1	3
5/12/2016	Outside	Dsuz	1	0	0
5/12/2016	Outside	Dsuz	3	1	1
5/12/2016	Outside	Dsuz	2	0	0
5/12/2016	Outside	Dsuz	3	1	1
5/12/2016	Outside	Dsuz	3	0	1
5/12/2016	Outside	Dsuz	2	0	1
5/12/2016	Outside	Dsuz	1	0	0
5/12/2016	Outside	Dsuz	1	0	0
5/12/2016	Outside	Dsuz	5	1	4
5/12/2016	Outside	Dsuz	4	1	1
5/12/2016	Outside	Dsuz	1	0	0
5/12/2016	Outside	Dsuz	1	0	0
5/12/2016	Outside	Dsuz	1	0	1
5/12/2016	Outside	Dsuz	9	3	3

5/12/2016	Outside	Dsuz	1	0	0
5/12/2016	Outside	Dsuz	4	2	1
5/12/2016	Outside	Dsuz	3	0	1
5/12/2016	Outside	Dsuz	1	0	0
5/12/2016	Outside	Dsuz	5	1	4
15/12/2016	Outside	Dsuz	5	0	3
15/12/2016	Outside	Dsuz	1	0	1
15/12/2016	Outside	Dsuz	1	0	0
15/12/2016	Outside	Dsuz	2	0	1
15/12/2016	Outside	Dsuz	1	0	1
15/12/2016	Outside	Dsuz	1	0	1
15/12/2016	Outside	Dsuz	2	0	1
15/12/2016	Outside	Dsuz	2	0	1
15/12/2016	Outside	Dsuz	4	1	1
15/12/2016	Outside	Dsuz	3	0	3
15/12/2016	Outside	Dsuz	1	0	1
15/12/2016	Outside	Dsuz	1	0	1
15/12/2016	Outside	Dsuz	2	0	1
15/12/2016	Outside	Dsuz	2	1	1
15/12/2016	Outside	Dsuz	4	1	1
15/12/2016	Outside	Dsuz	3	0	3
15/12/2016	Outside	Dsuz	2	0	1
15/12/2016	Outside	Dsuz	4	2	1
15/12/2016	Outside	Dsuz	3	1	1
15/12/2016	Outside	Dsuz	3	1	1
26/12/2016	Outside	Dsuz	12	0	6
26/12/2016	Outside	Dsuz	2	0	0
26/12/2016	Outside	Dsuz	10	4	2
26/12/2016	Outside	Dsuz	6	1	1
26/12/2016	Outside	Dsuz	5	0	0
26/12/2016	Outside	Dsuz	1	0	0
26/12/2016	Outside	Dsuz	9	1	2
26/12/2016	Outside	Dsuz	5	0	1
26/12/2016	Outside	Dsuz	12	3	5
26/12/2016	Outside	Dsuz	6	0	1
26/12/2016	Outside	Dsuz	4	0	2
26/12/2016	Outside	Dsuz	11	3	4
26/12/2016	Outside	Dsuz	2	0	0
26/12/2016	Outside	Dsuz	3	0	0
26/12/2016	Outside	Dsuz	8	2	2
26/12/2016	Outside	Dsuz	2	0	0
26/12/2016	Outside	Dsuz	6	1	1
26/12/2016	Outside	Dsuz	4	1	1
26/12/2016	Outside	Dsuz	3	0	1
26/12/2016	Outside	Dsuz	6	1	2
5/1/2017	Outside	Dsuz	2	1	1
5/1/2017	Outside	Dsuz	6	1	1

5/1/2017	Outside	Dsuz	8	3	2
5/1/2017	Outside	Dsuz	4	1	1
5/1/2017	Outside	Dsuz	5	1	1
5/1/2017	Outside	Dsuz	1	0	0
5/1/2017	Outside	Dsuz	3	1	0
5/1/2017	Outside	Dsuz	4	2	0
5/1/2017	Outside	Dsuz	5	1	2
5/1/2017	Outside	Dsuz	3	1	2
5/1/2017	Outside	Dsuz	2	1	0
5/1/2017	Outside	Dsuz	2	1	0
5/1/2017	Outside	Dsuz	1	0	0
5/1/2017	Outside	Dsuz	2	1	1
5/1/2017	Outside	Dsuz	1	0	0
5/1/2017	Outside	Dsuz	1	0	0
5/1/2017	Outside	Dsuz	2	0	1
5/1/2017	Outside	Dsuz	1	0	0
5/1/2017	Outside	Dsuz	3	1	1
5/1/2017	Outside	Dsuz	3	1	1
16/1/2017	Outside	Dsuz	7	1	2
16/1/2017	Outside	Dsuz	3	1	1
16/1/2017	Outside	Dsuz	3	0	1
16/1/2017	Outside	Dsuz	8	2	3
16/1/2017	Outside	Dsuz	5	1	2
16/1/2017	Outside	Dsuz	1	0	1
16/1/2017	Outside	Dsuz	2	0	1
16/1/2017	Outside	Dsuz	4	0	1
16/1/2017	Outside	Dsuz	8	1	1
16/1/2017	Outside	Dsuz	5	1	1
16/1/2017	Outside	Dsuz	3	0	1
16/1/2017	Outside	Dsuz	8	1	2
16/1/2017	Outside	Dsuz	4	0	1
16/1/2017	Outside	Dsuz	5	0	2
16/1/2017	Outside	Dsuz	2	0	1
16/1/2017	Outside	Dsuz	3	1	1
16/1/2017	Outside	Dsuz	2	0	1
16/1/2017	Outside	Dsuz	4	1	2
16/1/2017	Outside	Dsuz	5	0	1
16/1/2017	Outside	Dsuz	8	0	2
26/1/2017	Outside	Dsuz	3	0	1
26/1/2017	Outside	Dsuz	2	0	1
26/1/2017	Outside	Dsuz	4	1	1
26/1/2017	Outside	Dsuz	6	1	1
26/1/2017	Outside	Dsuz	3	1	0
26/1/2017	Outside	Dsuz	5	1	2
26/1/2017	Outside	Dsuz	12	1	5
26/1/2017	Outside	Dsuz	3	1	1
26/1/2017	Outside	Dsuz	2	0	0

26/1/2017	Outside	Dsuz	4	1	1
26/1/2017	Outside	Dsuz	5	1	1
26/1/2017	Outside	Dsuz	7	1	1
26/1/2017	Outside	Dsuz	2	0	1
26/1/2017	Outside	Dsuz	4	1	1
26/1/2017	Outside	Dsuz	6	1	1
26/1/2017	Outside	Dsuz	2	0	0
26/1/2017	Outside	Dsuz	7	1	1
26/1/2017	Outside	Dsuz	4	1	1
26/1/2017	Outside	Dsuz	3	0	1
26/1/2017	Outside	Dsuz	2	1	1
5/12/2016	Soil	Dsuz	1	0	1
5/12/2016	Soil	Dsuz	1	0	1
5/12/2016	Soil	Dsuz	1	0	1
5/12/2016	Soil	Dsuz	1	0	1
5/12/2016	Soil	Dsuz	2	0	1
5/12/2016	Soil	Dsuz	1	0	1
5/12/2016	Soil	Dsuz	2	0	1
5/12/2016	Soil	Dsuz	1	0	1
5/12/2016	Soil	Dsuz	2	0	1
5/12/2016	Soil	Dsuz	1	0	1
5/12/2016	Soil	Dsuz	2	0	1
5/12/2016	Soil	Dsuz	9	1	4
5/12/2016	Soil	Dsuz	6	0	3
5/12/2016	Soil	Dsuz	1	0	1
5/12/2016	Soil	Dsuz	5	0	1
5/12/2016	Soil	Dsuz	3	0	1
5/12/2016	Soil	Dsuz	3	0	1
5/12/2016	Soil	Dsuz	1	0	1
5/12/2016	Soil	Dsuz	2	0	1
5/12/2016	Soil	Dsuz	2	0	1
15/12/2016	Soil	Dsuz	1	0	0
15/12/2016	Soil	Dsuz	1	0	1
15/12/2016	Soil	Dsuz	2	0	1
15/12/2016	Soil	Dsuz	3	0	2
15/12/2016	Soil	Dsuz	3	0	3
15/12/2016	Soil	Dsuz	1	0	0
15/12/2016	Soil	Dsuz	0	0	0
15/12/2016	Soil	Dsuz	1	0	1
15/12/2016	Soil	Dsuz	1	0	1
15/12/2016	Soil	Dsuz	6	0	4
15/12/2016	Soil	Dsuz	1	0	1
15/12/2016	Soil	Dsuz	1	0	0
15/12/2016	Soil	Dsuz	1	0	1
15/12/2016	Soil	Dsuz	1	0	0
15/12/2016	Soil	Dsuz	1	0	0
15/12/2016	Soil	Dsuz	3	0	2

15/12/2016	Soil	Dsuz	1	0	1
15/12/2016	Soil	Dsuz	1	0	0
15/12/2016	Soil	Dsuz	2	0	2
15/12/2016	Soil	Dsuz	1	0	1
26/12/2016	Soil	Dsuz	2	0	1
26/12/2016	Soil	Dsuz	3	0	1
26/12/2016	Soil	Dsuz	5	1	2
26/12/2016	Soil	Dsuz	1	0	1
26/12/2016	Soil	Dsuz	4	0	3
26/12/2016	Soil	Dsuz	2	0	1
26/12/2016	Soil	Dsuz	3	0	1
26/12/2016	Soil	Dsuz	3	0	1
26/12/2016	Soil	Dsuz	6	0	3
26/12/2016	Soil	Dsuz	2	0	1
26/12/2016	Soil	Dsuz	3	0	1
26/12/2016	Soil	Dsuz	3	0	1
26/12/2016	Soil	Dsuz	5	0	2
26/12/2016	Soil	Dsuz	6	0	2
26/12/2016	Soil	Dsuz	3	0	2
26/12/2016	Soil	Dsuz	3	0	1
26/12/2016	Soil	Dsuz	3	0	2
26/12/2016	Soil	Dsuz	3	0	1
26/12/2016	Soil	Dsuz	2	0	1
26/12/2016	Soil	Dsuz	4	0	2
5/1/2017	Soil	Dsuz	2	0	1
5/1/2017	Soil	Dsuz	4	0	2
5/1/2017	Soil	Dsuz	2	0	1
5/1/2017	Soil	Dsuz	1	0	0
5/1/2017	Soil	Dsuz	1	0	0
5/1/2017	Soil	Dsuz	2	0	1
5/1/2017	Soil	Dsuz	1	0	0
5/1/2017	Soil	Dsuz	1	0	0
5/1/2017	Soil	Dsuz	2	0	1
5/1/2017	Soil	Dsuz	6	0	4
5/1/2017	Soil	Dsuz	1	0	1
5/1/2017	Soil	Dsuz	1	0	0
5/1/2017	Soil	Dsuz	1	0	0
5/1/2017	Soil	Dsuz	1	0	1
5/1/2017	Soil	Dsuz	2	0	1
5/1/2017	Soil	Dsuz	2	0	1
5/1/2017	Soil	Dsuz	2	0	1
5/1/2017	Soil	Dsuz	2	0	1
5/1/2017	Soil	Dsuz	3	0	1
5/1/2017	Soil	Dsuz	2	0	2
16/1/2017	Soil	Dsuz	4	0	1
16/1/2017	Soil	Dsuz	2	0	1
16/1/2017	Soil	Dsuz	2	0	1

16/1/2017	Soil	Dsuz	3	1	2
16/1/2017	Soil	Dsuz	3	1	2
16/1/2017	Soil	Dsuz	1	0	1
16/1/2017	Soil	Dsuz	1	0	0
16/1/2017	Soil	Dsuz	2	0	1
16/1/2017	Soil	Dsuz	2	0	1
16/1/2017	Soil	Dsuz	2	0	1
16/1/2017	Soil	Dsuz	2	0	1
16/1/2017	Soil	Dsuz	3	0	1
16/1/2017	Soil	Dsuz	2	0	1
16/1/2017	Soil	Dsuz	2	0	1
16/1/2017	Soil	Dsuz	4	0	2
16/1/2017	Soil	Dsuz	2	0	1
16/1/2017	Soil	Dsuz	4	0	1
16/1/2017	Soil	Dsuz	3	0	1
16/1/2017	Soil	Dsuz	4	0	2
16/1/2017	Soil	Dsuz	6	0	3
26/1/2017	Soil	Dsuz	2	0	0
26/1/2017	Soil	Dsuz	3	0	1
26/1/2017	Soil	Dsuz	2	0	1
26/1/2017	Soil	Dsuz	1	0	0
26/1/2017	Soil	Dsuz	1	0	0
26/1/2017	Soil	Dsuz	2	0	1
26/1/2017	Soil	Dsuz	3	0	1
26/1/2017	Soil	Dsuz	2	0	1
26/1/2017	Soil	Dsuz	5	0	2
26/1/2017	Soil	Dsuz	2	0	1
26/1/2017	Soil	Dsuz	3	0	0
26/1/2017	Soil	Dsuz	2	0	2
26/1/2017	Soil	Dsuz	2	0	1
26/1/2017	Soil	Dsuz	2	0	1
26/1/2017	Soil	Dsuz	7	0	3
26/1/2017	Soil	Dsuz	5	0	1
26/1/2017	Soil	Dsuz	3	0	1
26/1/2017	Soil	Dsuz	2	0	1
26/1/2017	Soil	Dsuz	9	0	3
26/1/2017	Soil	Dsuz	5	0	2
5/12/2016	Inside	Dspp	8	1	0
5/12/2016	Inside	Dspp	12	3	0
5/12/2016	Inside	Dspp	5	1	0
5/12/2016	Inside	Dspp	7	2	0
5/12/2016	Inside	Dspp	10	2	0
5/12/2016	Inside	Dspp	5	1	0
5/12/2016	Inside	Dspp	14	6	2
5/12/2016	Inside	Dspp	12	4	0
5/12/2016	Inside	Dspp	5	1	0
5/12/2016	Inside	Dspp	10	4	0

5/12/2016	Inside	Dspp	8	1	0
5/12/2016	Inside	Dspp	21	7	1
5/12/2016	Inside	Dspp	16	4	2
5/12/2016	Inside	Dspp	7	1	0
5/12/2016	Inside	Dspp	5	2	0
5/12/2016	Inside	Dspp	11	3	1
5/12/2016	Inside	Dspp	15	3	2
5/12/2016	Inside	Dspp	9	1	0
5/12/2016	Inside	Dspp	10	4	1
5/12/2016	Inside	Dspp	17	1	0
15/12/2016	Inside	Dspp	18	7	2
15/12/2016	Inside	Dspp	11	4	1
15/12/2016	Inside	Dspp	13	2	2
15/12/2016	Inside	Dspp	16	5	2
15/12/2016	Inside	Dspp	9	1	0
15/12/2016	Inside	Dspp	10	3	1
15/12/2016	Inside	Dspp	8	1	1
15/12/2016	Inside	Dspp	14	3	2
15/12/2016	Inside	Dspp	11	5	1
15/12/2016	Inside	Dspp	9	1	0
15/12/2016	Inside	Dspp	12	4	0
15/12/2016	Inside	Dspp	14	5	2
15/12/2016	Inside	Dspp	15	4	2
15/12/2016	Inside	Dspp	11	6	1
15/12/2016	Inside	Dspp	10	2	0
15/12/2016	Inside	Dspp	16	6	1
15/12/2016	Inside	Dspp	23	10	3
15/12/2016	Inside	Dspp	14	5	1
15/12/2016	Inside	Dspp	19	7	2
15/12/2016	Inside	Dspp	13	5	1
26/12/2016	Inside	Dspp	17	4	1
26/12/2016	Inside	Dspp	14	5	1
26/12/2016	Inside	Dspp	23	11	2
26/12/2016	Inside	Dspp	10	2	0
26/12/2016	Inside	Dspp	16	5	0
26/12/2016	Inside	Dspp	19	3	0
26/12/2016	Inside	Dspp	21	9	0
26/12/2016	Inside	Dspp	27	7	2
26/12/2016	Inside	Dspp	15	6	2
26/12/2016	Inside	Dspp	14	7	1
26/12/2016	Inside	Dspp	13	6	0
26/12/2016	Inside	Dspp	11	5	0
26/12/2016	Inside	Dspp	18	7	0
26/12/2016	Inside	Dspp	26	13	3
26/12/2016	Inside	Dspp	19	8	1
26/12/2016	Inside	Dspp	12	7	0
26/12/2016	Inside	Dspp	15	5	1

26/12/2016	Inside	Dspp	14	4	1
26/12/2016	Inside	Dspp	10	3	0
26/12/2016	Inside	Dspp	13	3	1
5/1/2017	Inside	Dspp	12	3	0
5/1/2017	Inside	Dspp	13	4	0
5/1/2017	Inside	Dspp	10	2	0
5/1/2017	Inside	Dspp	9	3	0
5/1/2017	Inside	Dspp	15	5	0
5/1/2017	Inside	Dspp	23	9	1
5/1/2017	Inside	Dspp	8	2	0
5/1/2017	Inside	Dspp	12	7	0
5/1/2017	Inside	Dspp	11	4	0
5/1/2017	Inside	Dspp	13	5	0
5/1/2017	Inside	Dspp	21	9	0
5/1/2017	Inside	Dspp	12	4	0
5/1/2017	Inside	Dspp	14	5	0
5/1/2017	Inside	Dspp	18	4	0
5/1/2017	Inside	Dspp	14	6	0
5/1/2017	Inside	Dspp	22	8	0
5/1/2017	Inside	Dspp	17	3	0
5/1/2017	Inside	Dspp	14	2	0
5/1/2017	Inside	Dspp	19	3	0
5/1/2017	Inside	Dspp	26	12	0
16/1/2017	Inside	Dspp	22	9	0
16/1/2017	Inside	Dspp	10	3	0
16/1/2017	Inside	Dspp	15	2	0
16/1/2017	Inside	Dspp	12	3	1
16/1/2017	Inside	Dspp	14	5	0
16/1/2017	Inside	Dspp	17	4	0
16/1/2017	Inside	Dspp	21	7	0
16/1/2017	Inside	Dspp	14	5	0
16/1/2017	Inside	Dspp	10	3	0
16/1/2017	Inside	Dspp	12	3	2
16/1/2017	Inside	Dspp	13	5	0
16/1/2017	Inside	Dspp	10	3	0
16/1/2017	Inside	Dspp	12	7	0
16/1/2017	Inside	Dspp	18	5	1
16/1/2017	Inside	Dspp	25	9	0
16/1/2017	Inside	Dspp	9	1	0
16/1/2017	Inside	Dspp	12	3	0
16/1/2017	Inside	Dspp	23	6	0
16/1/2017	Inside	Dspp	15	3	0
16/1/2017	Inside	Dspp	17	3	0
26/1/2017	Inside	Dspp	9	3	0
26/1/2017	Inside	Dspp	15	4	0
26/1/2017	Inside	Dspp	14	3	0
26/1/2017	Inside	Dspp	16	5	0

26/1/2017	Inside	Dspp	13	4	0
26/1/2017	Inside	Dspp	13	4	0
26/1/2017	Inside	Dspp	18	7	0
26/1/2017	Inside	Dspp	14	3	2
26/1/2017	Inside	Dspp	16	3	0
26/1/2017	Inside	Dspp	17	2	0
26/1/2017	Inside	Dspp	19	7	0
26/1/2017	Inside	Dspp	14	8	1
26/1/2017	Inside	Dspp	34	11	0
26/1/2017	Inside	Dspp	22	9	0
26/1/2017	Inside	Dspp	13	5	0
26/1/2017	Inside	Dspp	10	4	0
26/1/2017	Inside	Dspp	31	6	1
26/1/2017	Inside	Dspp	26	5	0
26/1/2017	Inside	Dspp	24	8	1
26/1/2017	Inside	Dspp	18	3	0
5/12/2016	Outside	Dspp	3	1	0
5/12/2016	Outside	Dspp	5	1	0
5/12/2016	Outside	Dspp	3	1	0
5/12/2016	Outside	Dspp	6	2	1
5/12/2016	Outside	Dspp	5	0	0
5/12/2016	Outside	Dspp	4	0	0
5/12/2016	Outside	Dspp	5	0	3
5/12/2016	Outside	Dspp	3	0	1
5/12/2016	Outside	Dspp	6	1	0
5/12/2016	Outside	Dspp	1	0	0
5/12/2016	Outside	Dspp	5	1	1
5/12/2016	Outside	Dspp	8	3	1
5/12/2016	Outside	Dspp	3	1	0
5/12/2016	Outside	Dspp	6	1	1
5/12/2016	Outside	Dspp	4	1	1
5/12/2016	Outside	Dspp	4	1	0
5/12/2016	Outside	Dspp	3	0	0
5/12/2016	Outside	Dspp	2	2	0
5/12/2016	Outside	Dspp	5	1	1
5/12/2016	Outside	Dspp	7	0	1
15/12/2016	Outside	Dspp	6	2	1
15/12/2016	Outside	Dspp	5	1	1
15/12/2016	Outside	Dspp	4	1	1
15/12/2016	Outside	Dspp	9	2	3
15/12/2016	Outside	Dspp	11	7	1
15/12/2016	Outside	Dspp	5	2	2
15/12/2016	Outside	Dspp	4	0	0
15/12/2016	Outside	Dspp	5	1	1
15/12/2016	Outside	Dspp	7	2	0
15/12/2016	Outside	Dspp	3	0	0
15/12/2016	Outside	Dspp	5	1	0

15/12/2016	Outside	Dspp	6	1	1
15/12/2016	Outside	Dspp	9	2	1
15/12/2016	Outside	Dspp	7	2	2
15/12/2016	Outside	Dspp	5	1	0
15/12/2016	Outside	Dspp	8	3	2
15/12/2016	Outside	Dspp	9	3	2
15/12/2016	Outside	Dspp	8	2	0
15/12/2016	Outside	Dspp	14	5	2
15/12/2016	Outside	Dspp	7	0	2
26/12/2016	Outside	Dspp	9	0	3
26/12/2016	Outside	Dspp	7	1	1
26/12/2016	Outside	Dspp	6	0	2
26/12/2016	Outside	Dspp	7	1	1
26/12/2016	Outside	Dspp	9	2	1
26/12/2016	Outside	Dspp	10	0	7
26/12/2016	Outside	Dspp	9	1	2
26/12/2016	Outside	Dspp	7	2	2
26/12/2016	Outside	Dspp	19	2	10
26/12/2016	Outside	Dspp	9	3	2
26/12/2016	Outside	Dspp	6	0	1
26/12/2016	Outside	Dspp	6	1	1
26/12/2016	Outside	Dspp	7	2	3
26/12/2016	Outside	Dspp	11	3	5
26/12/2016	Outside	Dspp	6	0	3
26/12/2016	Outside	Dspp	9	2	2
26/12/2016	Outside	Dspp	12	2	5
26/12/2016	Outside	Dspp	4	0	2
26/12/2016	Outside	Dspp	9	2	2
26/12/2016	Outside	Dspp	6	2	2
5/1/2017	Outside	Dspp	7	2	1
5/1/2017	Outside	Dspp	11	6	1
5/1/2017	Outside	Dspp	9	2	1
5/1/2017	Outside	Dspp	8	2	1
5/1/2017	Outside	Dspp	6	2	1
5/1/2017	Outside	Dspp	15	6	4
5/1/2017	Outside	Dspp	9	2	1
5/1/2017	Outside	Dspp	6	2	1
5/1/2017	Outside	Dspp	4	2	0
5/1/2017	Outside	Dspp	8	3	1
5/1/2017	Outside	Dspp	8	2	1
5/1/2017	Outside	Dspp	7	3	1
5/1/2017	Outside	Dspp	8	2	1
5/1/2017	Outside	Dspp	5	2	1
5/1/2017	Outside	Dspp	9	2	1
5/1/2017	Outside	Dspp	8	3	1
5/1/2017	Outside	Dspp	7	2	1
5/1/2017	Outside	Dspp	13	3	6

5/1/2017	Outside	Dspp	6	3	1
5/1/2017	Outside	Dspp	12	6	3
16/1/2017	Outside	Dspp	10	6	0
16/1/2017	Outside	Dspp	9	1	0
16/1/2017	Outside	Dspp	4	1	0
16/1/2017	Outside	Dspp	7	1	1
16/1/2017	Outside	Dspp	6	1	1
16/1/2017	Outside	Dspp	3	0	0
16/1/2017	Outside	Dspp	12	4	0
16/1/2017	Outside	Dspp	3	0	0
16/1/2017	Outside	Dspp	4	1	0
16/1/2017	Outside	Dspp	7	1	1
16/1/2017	Outside	Dspp	8	2	0
16/1/2017	Outside	Dspp	9	2	1
16/1/2017	Outside	Dspp	3	0	0
16/1/2017	Outside	Dspp	7	0	1
16/1/2017	Outside	Dspp	12	3	0
16/1/2017	Outside	Dspp	3	1	0
16/1/2017	Outside	Dspp	8	2	0
16/1/2017	Outside	Dspp	18	5	1
16/1/2017	Outside	Dspp	11	6	0
16/1/2017	Outside	Dspp	9	3	1
26/1/2017	Outside	Dspp	4	1	1
26/1/2017	Outside	Dspp	4	1	2
26/1/2017	Outside	Dspp	5	2	3
26/1/2017	Outside	Dspp	5	2	1
26/1/2017	Outside	Dspp	6	1	2
26/1/2017	Outside	Dspp	5	2	1
26/1/2017	Outside	Dspp	3	0	1
26/1/2017	Outside	Dspp	4	2	2
26/1/2017	Outside	Dspp	4	1	1
26/1/2017	Outside	Dspp	3	0	1
26/1/2017	Outside	Dspp	8	2	2
26/1/2017	Outside	Dspp	5	2	2
26/1/2017	Outside	Dspp	12	1	3
26/1/2017	Outside	Dspp	10	2	5
26/1/2017	Outside	Dspp	8	2	3
26/1/2017	Outside	Dspp	5	1	2
26/1/2017	Outside	Dspp	8	1	2
26/1/2017	Outside	Dspp	5	1	3
26/1/2017	Outside	Dspp	7	1	3
26/1/2017	Outside	Dspp	6	1	2
5/12/2016	Soil	Dspp	1	0	1
5/12/2016	Soil	Dspp	1	0	0
5/12/2016	Soil	Dspp	0	0	0
5/12/2016	Soil	Dspp	0	0	0
5/12/2016	Soil	Dspp	0	0	0

5/12/2016	Soil	Dspp	0	0	0
5/12/2016	Soil	Dspp	0	0	0
5/12/2016	Soil	Dspp	0	0	0
5/12/2016	Soil	Dspp	0	0	0
5/12/2016	Soil	Dspp	0	0	0
5/12/2016	Soil	Dspp	0	0	0
5/12/2016	Soil	Dspp	0	0	0
5/12/2016	Soil	Dspp	0	0	0
5/12/2016	Soil	Dspp	0	0	0
5/12/2016	Soil	Dspp	0	0	0
5/12/2016	Soil	Dspp	0	0	0
5/12/2016	Soil	Dspp	0	0	0
5/12/2016	Soil	Dspp	0	0	0
5/12/2016	Soil	Dspp	0	0	0
5/12/2016	Soil	Dspp	0	0	0
15/12/2016	Soil	Dspp	0	0	0
15/12/2016	Soil	Dspp	0	0	0
15/12/2016	Soil	Dspp	0	0	0
15/12/2016	Soil	Dspp	1	0	0
15/12/2016	Soil	Dspp	1	0	0
15/12/2016	Soil	Dspp	0	0	0
15/12/2016	Soil	Dspp	0	0	0
15/12/2016	Soil	Dspp	1	0	0
15/12/2016	Soil	Dspp	0	0	0
15/12/2016	Soil	Dspp	1	0	0
15/12/2016	Soil	Dspp	0	0	0
15/12/2016	Soil	Dspp	1	0	0
15/12/2016	Soil	Dspp	0	0	0
15/12/2016	Soil	Dspp	1	0	1
15/12/2016	Soil	Dspp	0	0	0
15/12/2016	Soil	Dspp	0	0	0
15/12/2016	Soil	Dspp	1	1	0
15/12/2016	Soil	Dspp	0	0	0
15/12/2016	Soil	Dspp	1	0	0
15/12/2016	Soil	Dspp	0	0	0
26/12/2016	Soil	Dspp	1	0	0
26/12/2016	Soil	Dspp	1	0	0
26/12/2016	Soil	Dspp	2	1	1
26/12/2016	Soil	Dspp	1	0	0
26/12/2016	Soil	Dspp	2	1	1
26/12/2016	Soil	Dspp	1	0	1
26/12/2016	Soil	Dspp	1	0	0
26/12/2016	Soil	Dspp	2	0	1
26/12/2016	Soil	Dspp	1	0	0
26/12/2016	Soil	Dspp	1	0	0
26/12/2016	Soil	Dspp	1	0	0
26/12/2016	Soil	Dspp	1	0	0

26/12/2016	Soil	Dspp	2	0	1
26/12/2016	Soil	Dspp	3	0	0
26/12/2016	Soil	Dspp	1	0	0
26/12/2016	Soil	Dspp	1	0	0
26/12/2016	Soil	Dspp	2	0	0
26/12/2016	Soil	Dspp	1	0	0
26/12/2016	Soil	Dspp	1	0	0
26/12/2016	Soil	Dspp	1	0	0
5/1/2017	Soil	Dspp	0	0	0
5/1/2017	Soil	Dspp	0	0	0
5/1/2017	Soil	Dspp	0	0	0
5/1/2017	Soil	Dspp	0	0	0
5/1/2017	Soil	Dspp	0	0	0
5/1/2017	Soil	Dspp	1	1	0
5/1/2017	Soil	Dspp	0	0	0
5/1/2017	Soil	Dspp	0	0	0
5/1/2017	Soil	Dspp	0	0	0
5/1/2017	Soil	Dspp	0	0	0
5/1/2017	Soil	Dspp	0	0	0
5/1/2017	Soil	Dspp	0	0	0
5/1/2017	Soil	Dspp	0	0	0
5/1/2017	Soil	Dspp	0	0	0
5/1/2017	Soil	Dspp	0	0	0
5/1/2017	Soil	Dspp	0	0	0
5/1/2017	Soil	Dspp	0	0	0
5/1/2017	Soil	Dspp	1	0	0
5/1/2017	Soil	Dspp	0	0	0
5/1/2017	Soil	Dspp	1	0	1
16/1/2017	Soil	Dspp	2	1	1
16/1/2017	Soil	Dspp	1	0	0
16/1/2017	Soil	Dspp	0	0	0
16/1/2017	Soil	Dspp	1	0	1
16/1/2017	Soil	Dspp	0	0	0
16/1/2017	Soil	Dspp	0	0	0
16/1/2017	Soil	Dspp	1	0	0
16/1/2017	Soil	Dspp	1	0	1
16/1/2017	Soil	Dspp	0	0	0
16/1/2017	Soil	Dspp	1	0	1
16/1/2017	Soil	Dspp	1	0	0
16/1/2017	Soil	Dspp	1	0	0
16/1/2017	Soil	Dspp	0	0	0
16/1/2017	Soil	Dspp	0	0	0
16/1/2017	Soil	Dspp	2	0	1
16/1/2017	Soil	Dspp	0	0	0
16/1/2017	Soil	Dspp	1	0	1
16/1/2017	Soil	Dspp	2	0	1
16/1/2017	Soil	Dspp	1	0	0

16/1/2017	Soil	Dspp	0	0	0
26/1/2017	Soil	Dspp	1	0	0
26/1/2017	Soil	Dspp	0	0	0
26/1/2017	Soil	Dspp	1	0	0
26/1/2017	Soil	Dspp	0	0	0
26/1/2017	Soil	Dspp	0	0	0
26/1/2017	Soil	Dspp	1	0	1
26/1/2017	Soil	Dspp	1	0	0
26/1/2017	Soil	Dspp	0	0	0
26/1/2017	Soil	Dspp	0	0	0
26/1/2017	Soil	Dspp	1	0	1
26/1/2017	Soil	Dspp	1	0	0
26/1/2017	Soil	Dspp	0	0	0
26/1/2017	Soil	Dspp	1	0	0
26/1/2017	Soil	Dspp	1	0	0
26/1/2017	Soil	Dspp	0	0	0
26/1/2017	Soil	Dspp	1	0	1
26/1/2017	Soil	Dspp	1	0	0
26/1/2017	Soil	Dspp	1	0	0
26/1/2017	Soil	Dspp	1	0	1
26/1/2017	Soil	Dspp	0	0	0
