# Supplementary material for: Implications of the Niche Partitioning and Coexistence of Two Resident Parasitoids for Drosophila suzukii Management in Non-Crop Areas
Source: Insects. 2023 Feb 23;14(3):222. doi: 10.3390/insects14030222 (PMC10051101; doi:10.3390/insects14030222)
Supplement: Supplementary file 1 [file insects-14-00222-s001.zip › File S2 Aligned-Ranks-Transformation-ANOVA--Markdown---Guavas.pdf]

Aligned Rank Transform T. anastrephae & P. vindemiae in Guavas

Segundo Núñez Campero

2022-12-16

The analysis was performed using a Aligned Rang Transformation with the packages 'ARTool' [@wobbrock2011], a factorial model done in preparation for a nonparametric analysis of variance models with numeric or ordinal responses. This is done by first aligning and ranking the fixed effects using the model function, then conducting an analysis of variance on linear models built from the transformed data using the function 'anova.art'. A post hoc pairwise comparisons was conducted to show differences between factor levels, with a Bonferroni–Holm adjust method, using the 'art.con' function [@elkin2021]. And a the library 'rcompanion' was used for obtain the letters that show significant difference.

T. anastrephae analysis

Model, sums of aligned responses and ANOVAs on aligned responses not of interest (should all be ~0)

```
m8 <- art(tric_suc ~ hab + fly_sp + hab * fly_sp, data=dt)
summary(m8)
```

```
## Aligned Rank Transform of Factorial Model
##
## Call:
## art(formula = tric_suc ~ hab + fly_sp + hab * fly_sp, data = dt)
##
## Column sums of aligned responses (should all be ~0):
##        hab     fly_sp hab:fly_sp
##          0          0          0
##
## F values of ANOVAs on aligned responses not of interest (should all be ~0):
##    Min. 1st Qu.  Median    Mean 3rd Qu.    Max.
##       0       0       0       0       0       0
```

```
anova(m8, response="aligned")
```

```
## Analysis of Variance of Aligned Rank Transformed Data
##
## Table Type: Anova Table (Type III tests)
## Model: No Repeated Measures (lm)
## Response: aligned(tric_suc)
##
##               Aligned By Df Df.res F value Pr(>F)
## 1 fly_sp             hab  1    714       0      1
## 2 hab:fly_sp         hab  2    714       0      1
## 3 hab             fly_sp  2    714       0      1
## 4 hab:fly_sp      fly_sp  2    714       0      1
## 5 hab         hab:fly_sp  2    714       0      1
```

```
## 6 fly_sp     hab:fly_sp  1    714       0      1
## ---
## Signif. codes:   0 '***' 0.001 '**' 0.01 '*' 0.05 '.' 0.1 ' ' 1
```

```r
anova(m8)
```

```
## Analysis of Variance of Aligned Rank Transformed Data
##
## Table Type: Anova Table (Type III tests)
## Model: No Repeated Measures (lm)
## Response: art(tric_suc)
##
##              Df Df.res F value     Pr(>F)
## 1 hab         2    714  599.24 < 2.22e-16 ***
## 2 fly_sp      1    714  733.73 < 2.22e-16 ***
## 3 hab:fly_sp  2    714  298.26 < 2.22e-16 ***
## ---
## Signif. codes:   0 '***' 0.001 '**' 0.01 '*' 0.05 '.' 0.1 ' ' 1
```

Constrasts

```r
m9 <- art.con(m8, "hab:fly_sp", adjust="holm") %>%
  summary() %>%  # add significance stars to the output
  mutate(sig. = symnum(p.value, corr=FALSE, na=FALSE,
                       cutpoints = c(0, .001, .01, .05, .10, 1),
                       symbols = c("***", "**", "*", ".", " ")))
m9
```

Interaction contrast

```
##                      contrast    estimate       SE  df     t.ratio
## 1    Inside,Dsp - Inside,Dsuz  319.095833 12.81103 714  24.9078937
## 2    Inside,Dsp - Outside,Dsp  144.275000 12.81103 714  11.2617778
## 3   Inside,Dsp - Outside,Dsuz  355.770833 12.81103 714  27.7706606
## 4       Inside,Dsp - Soil,Dsp  428.608333 12.81103 714  33.4561899
## 5      Inside,Dsp - Soil,Dsuz  430.750000 12.81103 714  33.6233635
## 6   Inside,Dsuz - Outside,Dsp -174.820833 12.81103 714 -13.6461159
## 7  Inside,Dsuz - Outside,Dsuz   36.675000 12.81103 714   2.8627669
## 8      Inside,Dsuz - Soil,Dsp  109.512500 12.81103 714   8.5482962
## 9     Inside,Dsuz - Soil,Dsuz  111.654167 12.81103 714   8.7154698
## 10 Outside,Dsp - Outside,Dsuz  211.495833 12.81103 714  16.5088828
## 11     Outside,Dsp - Soil,Dsp  284.333333 12.81103 714  22.1944121
## 12    Outside,Dsp - Soil,Dsuz  286.475000 12.81103 714  22.3615858
## 13    Outside,Dsuz - Soil,Dsp   72.837500 12.81103 714   5.6855293
## 14   Outside,Dsuz - Soil,Dsuz   74.979167 12.81103 714   5.8527029
## 15        Soil,Dsp - Soil,Dsuz    2.141667 12.81103 714   0.1671736
##          p.value sig.
## 1   5.787768e-98  ***
## 2   2.383984e-26  ***
## 3  1.488219e-114  ***
## 4  3.328926e-147  ***
## 5  4.036081e-148  ***
## 6   6.113573e-37  ***
## 7   8.645265e-03   **
## 8   3.776838e-16  ***
```

```
## 9   1.206233e-16  ***
## 10  3.782302e-51  ***
## 11  2.097943e-82  ***
## 12  2.553287e-83  ***
## 13  5.703945e-08  ***
## 14  2.948194e-08  ***
## 15  8.672808e-01
```

```
Sum = as.data.frame(m9)
cldList(p.value ~ contrast, data=Sum) ### alfa level =  0.05
```

```
##          Group Letter MonoLetter
## 1   Inside,Dsp      a      a
## 2  Inside,Dsuz      b       b
## 3  Outside,Dsp      c        c
## 4 Outside,Dsuz      d         d
## 5     Soil,Dsp      e          e
## 6    Soil,Dsuz      e          e
```

```

m10 <- art.con(m8, "hab", adjust="holm") %>%
  summary() %>%  # add significance stars to the output
  mutate(sig. = symnum(p.value, corr=FALSE, na=FALSE,
                       cutpoints = c(0, .001, .01, .05, .10, 1),
                       symbols = c("***", "**", "*", ".", " ")))
```

Habitat Contrast

```
## NOTE: Results may be misleading due to involvement in interactions
```

```
m10
```

```
##           contrast estimate       SE  df  t.ratio       p.value sig.
## 1 Inside - Outside 158.1792 11.29202 714 14.00805  1.445595e-39  ***
## 2    Inside - Soil 388.6833 11.29202 714 34.42106 2.549974e-153  ***
## 3   Outside - Soil 230.5042 11.29202 714 20.41301  5.226034e-73  ***
```

```
Sum = as.data.frame(m10)
cldList(p.value ~ contrast, data=Sum) ### alfa level =  0.05
```

```
##     Group Letter MonoLetter
## 1  Inside      a         a
## 2 Outside      b          b
## 3    Soil      c           c
```

Fly species contrast

```
m11 <- art.con(m8, "fly_sp", adjust="holm") %>%
  summary() %>%  # add significance stars to the output
  mutate(sig. = symnum(p.value, corr=FALSE, na=FALSE,
                       cutpoints = c(0, .001, .01, .05, .10, 1),
                       symbols = c("***", "**", "*", ".", " ")))
```

```
## NOTE: Results may be misleading due to involvement in interactions
```

```
m11
```

```
##     contrast estimate       SE  df  t.ratio       p.value sig.
```

```
## 1 Dsp - Dsuz 290.7722 10.73461 714 27.08736 1.064715e-111  ***
```

```
Sum = as.data.frame(m11)
cldList(p.value ~ contrast, data=Sum) ### alfa level =  0.05
```

```
##   Group Letter MonoLetter
## 1   Dsp      a          a
## 2  Dsuz      b          b
```

Graphic of *T. anastrephae* success with interactions.

```
ggplot(dt, aes(x = factor(hab), y = tric_suc, fill = fly_sp, colour = fly_sp)) +
  geom_violin(trim = T, position=position_dodge(1), scale = "width") +
  scale_y_continuous(breaks=seq(0, 14.5, 2)) +
  labs(x = "Habitat",
       y = italic('Trichopria anastrephae')~ "success in guava") + #Axes title
  annotate('text', label='a',
           x=0.77, y=14.5, hjust=0.1, vjust=0, size=4, color='black') +
  annotate('text', label='c',
           x=1.22, y=5.5, hjust=0.1, vjust=0, size=4, color='black') +
  annotate('text', label='b',
           x=1.77, y=8.5, hjust=0.1, vjust=0, size=4, color='black') +
  annotate('text', label='d',
           x=2.22, y=5.5, hjust=0.1, vjust=0, size=4, color='black') +
  annotate('text', label='e',
           x=2.77, y=1.5, hjust=0.1, vjust=0, size=4, color='black') +
  annotate('text', label='e',
           x=3.20, y=1.5, hjust=0.1, vjust=0, size=4, color='black') +
  geom_boxplot(width=0.1, color="black", alpha=0.2, position=position_dodge(1)) +
  geom_vline(xintercept = 1.5) +
  geom_vline(xintercept = 2.5)
```

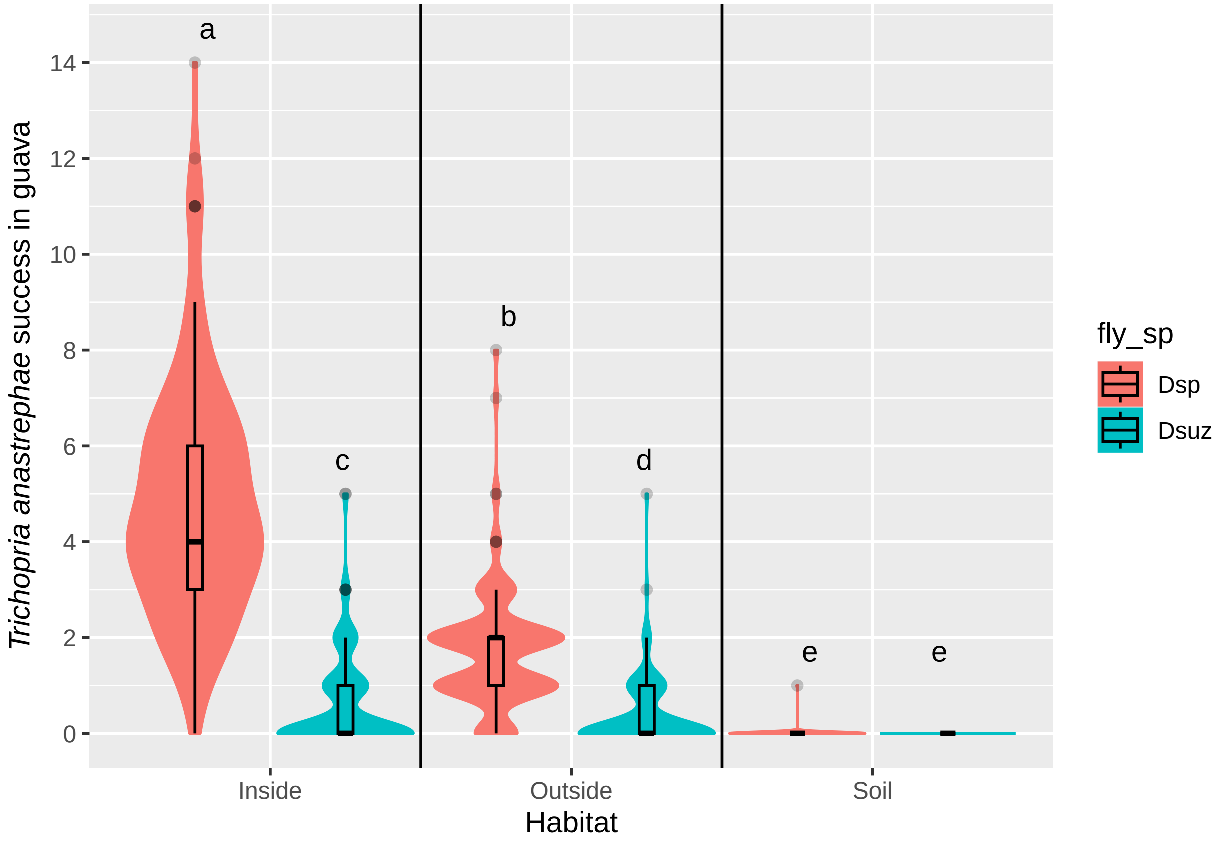

P. vindemiae analysis

Model, sums of aligned responses and ANOVAs on aligned responses not of interest (should all be ~0)

```
m12 <- art(pach_suc ~ hab + fly_sp + hab * fly_sp, data=dt)
summary(m12)
```

```
## Aligned Rank Transform of Factorial Model
##
## Call:
## art(formula = pach_suc ~ hab + fly_sp + hab * fly_sp, data = dt)
##
## Column sums of aligned responses (should all be ~0):
##        hab     fly_sp hab:fly_sp
##          0          0          0
##
## F values of ANOVAs on aligned responses not of interest (should all be ~0):
##    Min. 1st Qu.  Median    Mean 3rd Qu.    Max.
##       0       0       0       0       0       0
```

```
anova(m12, response="aligned")
```

```
## Analysis of Variance of Aligned Rank Transformed Data
##
```

```
## Table Type: Anova Table (Type III tests)
## Model: No Repeated Measures (lm)
## Response: aligned(pach_suc)
##
##              Aligned By Df Df.res F value Pr(>F)
## 1 fly_sp            hab  1    714       0      1
## 2 hab:fly_sp        hab  2    714       0      1
## 3 hab            fly_sp  2    714       0      1
## 4 hab:fly_sp     fly_sp  2    714       0      1
## 5 hab        hab:fly_sp  2    714       0      1
## 6 fly_sp     hab:fly_sp  1    714       0      1
## ---
## Signif. codes:   0 '***' 0.001 '**' 0.01 '*' 0.05 '.' 0.1 ' ' 1
```

```
anova(m12)
```

```
## Analysis of Variance of Aligned Rank Transformed Data
##
## Table Type: Anova Table (Type III tests)
## Model: No Repeated Measures (lm)
## Response: art(pach_suc)
##
##              Df Df.res F value     Pr(>F)
## 1 hab         2    714  52.673 < 2.22e-16 ***
## 2 fly_sp      1    714 102.911 < 2.22e-16 ***
## 3 hab:fly_sp  2    714  42.630 < 2.22e-16 ***
## ---
## Signif. codes:   0 '***' 0.001 '**' 0.01 '*' 0.05 '.' 0.1 ' ' 1
```

Contrasts

```
m13 <- art.con(m12, "hab:fly_sp", adjust="holm") %>%
  summary() %>%  # add significance stars to the output
  mutate(sig. = symnum(p.value, corr=FALSE, na=FALSE,
                       cutpoints = c(0, .001, .01, .05, .10, 1),
                       symbols = c("***", "**", "*", ".", " ")))
m13
```

Interaction contrast

```
##                      contrast   estimate       SE  df    t.ratio      p.value
## 1    Inside,Dsp - Inside,Dsuz   24.88333 19.23198 714  1.2938521 5.884046e-01
## 2    Inside,Dsp - Outside,Dsp -160.12500 19.23198 714 -8.3259774 5.958678e-15
## 3   Inside,Dsp - Outside,Dsuz  -60.70000 19.23198 714 -3.1562019 1.332443e-02
## 4       Inside,Dsp - Soil,Dsp  -14.08333 19.23198 714 -0.7322874 9.284670e-01
## 5      Inside,Dsp - Soil,Dsuz  -48.57500 19.23198 714 -2.5257415 8.232468e-02
## 6   Inside,Dsuz - Outside,Dsp -185.00833 19.23198 714 -9.6198295 1.664225e-19
## 7  Inside,Dsuz - Outside,Dsuz  -85.58333 19.23198 714 -4.4500540 9.956797e-05
## 8      Inside,Dsuz - Soil,Dsp  -38.96667 19.23198 714 -2.0261395 2.156091e-01
## 9     Inside,Dsuz - Soil,Dsuz  -73.45833 19.23198 714 -3.8195936 1.307705e-03
## 10 Outside,Dsp - Outside,Dsuz   99.42500 19.23198 714  5.1697755 3.350826e-06
## 11      Outside,Dsp - Soil,Dsp  146.04167 19.23198 714  7.5936900 1.268489e-12
## 12     Outside,Dsp - Soil,Dsuz  111.55000 19.23198 714  5.8002359 1.193838e-07
## 13     Outside,Dsuz - Soil,Dsp   46.61667 19.23198 714  2.4239145 9.360973e-02
## 14    Outside,Dsuz - Soil,Dsuz   12.12500 19.23198 714  0.6304604 9.284670e-01
```

```
## 15       Soil,Dsp - Soil,Dsuz  -34.49167 19.23198 714 -1.7934541 2.932937e-01
##    sig.
## 1
## 2   ***
## 3     *
## 4
## 5     .
## 6   ***
## 7   ***
## 8
## 9    **
## 10  ***
## 11  ***
## 12  ***
## 13    .
## 14
## 15
```

```
Sum = as.data.frame(m13)
cldList(p.value ~ contrast, data=Sum) ### alfa level =  0.05
```

```
##          Group Letter MonoLetter
## 1   Inside,Dsp     ab       ab
## 2  Inside,Dsuz      a       a
## 3  Outside,Dsp      c         c
## 4 Outside,Dsuz      d          d
## 5     Soil,Dsp    abd       ab d
## 6    Soil,Dsuz     bd        b d
```

```
m14 <- art.con(m12, "hab", adjust="holm") %>%
  summary() %>%  # add significance stars to the output
  mutate(sig. = symnum(p.value, corr=FALSE, na=FALSE,
                       cutpoints = c(0, .001, .01, .05, .10, 1),
                       symbols = c("***", "**", "*", ".", " ")))
```

Habitat Contrast

```
## NOTE: Results may be misleading due to involvement in interactions
```

```
m14
```

```
##           contrast   estimate       SE  df    t.ratio      p.value sig.
## 1 Inside - Outside -163.28333 16.17552 714 -10.094470 5.198033e-22  ***
## 2    Inside - Soil  -55.62917 16.17552 714  -3.439095 6.175610e-04  ***
## 3   Outside - Soil  107.65417 16.17552 714   6.655375 1.126323e-10  ***
```

```
Sum = as.data.frame(m14)
cldList(p.value ~ contrast, data=Sum) ### alfa level =  0.05
```

```
##     Group Letter MonoLetter
## 1  Inside      a        a
## 2 Outside      b         b
## 3    Soil      c          c
```

Fly species contrast

```
m15 <- art.con(m12, "fly_sp", adjust="holm") %>%
  summary() %>%  # add significance stars to the output
  mutate(sig. = symnum(p.value, corr=FALSE, na=FALSE,
                       cutpoints = c(0, .001, .01, .05, .10, 1),
                       symbols = c("***", "**", "*", ".", " ")))
```

```
## NOTE: Results may be misleading due to involvement in interactions
```

```
m15
```

```
##     contrast estimate       SE  df  t.ratio    p.value sig.
## 1 Dsp - Dsuz 142.7833 14.07492 714 10.14452 1.1079e-22  ***
```

```
Sum = as.data.frame(m15)
cldList(p.value ~ contrast, data=Sum) ### alfa level =  0.05
```

```
##   Group Letter MonoLetter
## 1   Dsp      a          a
## 2  Dsuz      b           b
```

Graphic of *P. vindemiae* success with interactions

```
ggplot(dt, aes(x = factor(hab), y = pach_suc, fill = fly_sp, colour = fly_sp)) +
  geom_violin(trim = T, position=position_dodge(1), scale = "width") +
  scale_y_continuous(breaks=seq(0, 5, 2)) +
  labs(x = "Habitat",
       y = italic('Pachycrepoideus vindemiae')~ "success in guava") +
  annotate('text', label='cd',
           x=0.75, y=3.25, hjust=0.1, vjust=0, size=4, color='black') +
  annotate('text', label='c',
           x=1.23, y=2.25, hjust=0.1, vjust=0, size=4, color='black') +
  annotate('text', label='a',
           x=1.75, y=5.25, hjust=0.1, vjust=0, size=4, color='black') +
  annotate('text', label='b',
           x=2.23, y=4.25, hjust=0.1, vjust=0, size=4, color='black') +
  annotate('text', label='bcd',
           x=2.70, y=2.25, hjust=0.1, vjust=0, size=4, color='black') +
  annotate('text', label='bd',
           x=3.20, y=3.25, hjust=0.1, vjust=0, size=4, color='black') +
  geom_boxplot(width=0.1, color="black", alpha=0.2, position=position_dodge(1)) +
  geom_vline(xintercept = 1.5) +
  geom_vline(xintercept = 2.5)
```

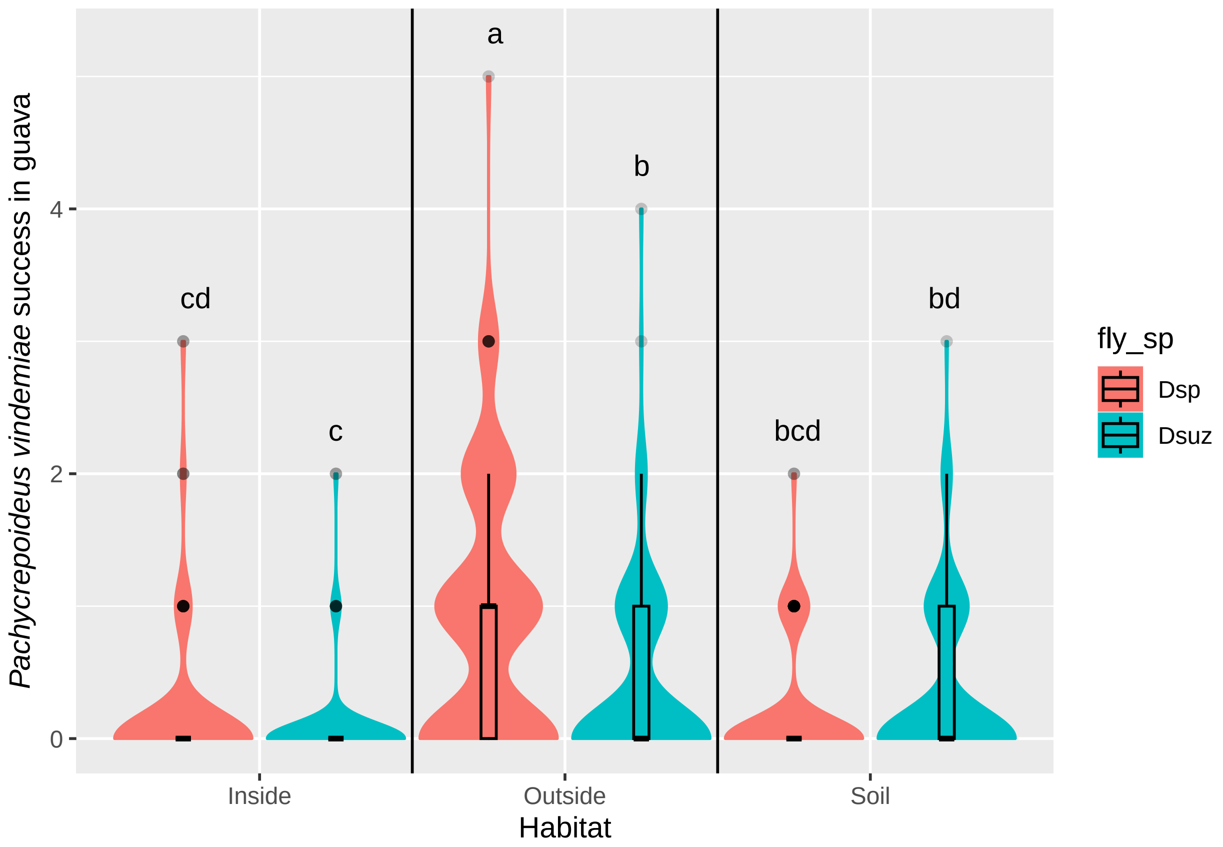
a
b
cd
c
bcd
bd
0
2
4
Pachycrepoideus vindemiae success in guava
Inside
Outside
Soil
Habitat
fly_sp
Dsp
Dsuz
