# Supplementary material for: Implications of the Niche Partitioning and Coexistence of Two Resident Parasitoids for Drosophila suzukii Management in Non-Crop Areas
Source: Insects. 2023 Feb 23;14(3):222. doi: 10.3390/insects14030222 (PMC10051101; doi:10.3390/insects14030222)
Supplement: Supplementary file 1 [file insects-14-00222-s001.zip › File S3 Aligned-Ranks-Transformation-ANOVA--Markdown---Peach.pdf]

Aligned Rank Transform T. anastrephae & P. vindemmiae on Peach

Segundo Núñez Campero

2022-12-16

The analysis was performed using a Aligned Rang Transformation with the packages 'ARTool' (Wobbrock et al. 2011), a factorial model done in preparation for a nonparametric analysis of variance models with numeric or ordinal responses. This is done by first aligning and ranking the fixed effects using the model function, then conducting an analysis of variance on linear models built from the transformed data using the function 'anova.art'. A *post hoc* pairwise comparisons was conducted to show differences between factor levels, with a Bonferroni–Holm adjust method, using the 'art.con' function (Elkin et al. 2021). And a the library 'rcompanion' was used for obtain the letters that show significant difference.

T. anastrephae **analysis**

Model, sums of aligned responses and ANOVAs on aligned responses not of interest (should all be ~0)

```
m <- art(tric_suc ~ hab + fly_sp + hab * fly_sp, data=dt)
summary(m)
```

```
## Aligned Rank Transform of Factorial Model
##
## Call:
## art(formula = tric_suc ~ hab + fly_sp + hab * fly_sp, data = dt)
##
## Column sums of aligned responses (should all be ~0):
##        hab     fly_sp hab:fly_sp
##          0          0          0
##
## F values of ANOVAs on aligned responses not of interest (should all be ~0):
##    Min. 1st Qu.  Median    Mean 3rd Qu.    Max.
##       0       0       0       0       0       0
```

```
anova(m, response="aligned")
```

```
## Analysis of Variance of Aligned Rank Transformed Data
##
## Table Type: Anova Table (Type III tests)
## Model: No Repeated Measures (lm)
## Response: aligned(tric_suc)
##
##              Aligned By Df Df.res F value Pr(>F)
## 1 fly_sp            hab  1    714       0      1
## 2 hab:fly_sp        hab  2    714       0      1
## 3 hab            fly_sp  2    714       0      1
## 4 hab:fly_sp     fly_sp  2    714       0      1
## 5 hab        hab:fly_sp  2    714       0      1
```

```
## 6 fly_sp     hab:fly_sp  1    714       0      1
## ---
## Signif. codes:   0 '***' 0.001 '**' 0.01 '*' 0.05 '.' 0.1 ' ' 1
```

```
anova(m)
```

```
## Analysis of Variance of Aligned Rank Transformed Data
##
## Table Type: Anova Table (Type III tests)
## Model: No Repeated Measures (lm)
## Response: art(tric_suc)
##
##               Df Df.res F value     Pr(>F)
## 1 hab          2    714  566.19 < 2.22e-16 ***
## 2 fly_sp       1    714  389.58 < 2.22e-16 ***
## 3 hab:fly_sp  2    714  204.23 < 2.22e-16 ***
## ---
## Signif. codes:   0 '***' 0.001 '**' 0.01 '*' 0.05 '.' 0.1 ' ' 1
```

Constrasts

```
m1 <- art.con(m, "hab:fly_sp", adjust="holm") %>%
  summary() %>%  # add significance stars to the output
  mutate(sig. = symnum(p.value, corr=FALSE, na=FALSE,
                       cutpoints = c(0, .001, .01, .05, .10, 1),
                       symbols = c("***", "**", "*", ".", " ")))
m1
```

Interaction contrast

```
##                      contrast   estimate       SE  df    t.ratio       p.value
## 1    Inside,Dsp - Inside,Dsuz 200.791667 14.37258 714 13.9704716  1.750721e-38
## 2    Inside,Dsp - Outside,Dsp 174.225000 14.37258 714 12.1220440  4.844841e-30
## 3   Inside,Dsp - Outside,Dsuz 322.529167 14.37258 714 22.4405954  1.065005e-83
## 4       Inside,Dsp - Soil,Dsp 451.691667 14.37258 714 31.4273281 1.218685e-135
## 5      Inside,Dsp - Soil,Dsuz 453.787500 14.37258 714 31.5731498 1.906506e-136
## 6   Inside,Dsuz - Outside,Dsp -26.566667 14.37258 714 -1.8484276  1.299076e-01
## 7  Inside,Dsuz - Outside,Dsuz 121.737500 14.37258 714  8.4701238  4.179134e-16
## 8      Inside,Dsuz - Soil,Dsp 250.900000 14.37258 714 17.4568565  3.812415e-56
## 9     Inside,Dsuz - Soil,Dsuz 252.995833 14.37258 714 17.6026782  7.052360e-57
## 10 Outside,Dsp - Outside,Dsuz 148.304167 14.37258 714 10.3185515  1.386529e-22
## 11     Outside,Dsp - Soil,Dsp 277.466667 14.37258 714 19.3052841  4.241522e-66
## 12    Outside,Dsp - Soil,Dsuz 279.562500 14.37258 714 19.4511058  7.226850e-67
## 13    Outside,Dsuz - Soil,Dsp 129.162500 14.37258 714  8.9867327  8.996048e-18
## 14   Outside,Dsuz - Soil,Dsuz 131.258333 14.37258 714  9.1325544  3.390520e-18
## 15        Soil,Dsp - Soil,Dsuz   2.095833 14.37258 714  0.1458217  8.841033e-01
##    sig.
## 1   ***
## 2   ***
## 3   ***
## 4   ***
## 5   ***
## 6
## 7   ***
## 8   ***
```

```
## 9   ***
## 10  ***
## 11  ***
## 12  ***
## 13  ***
## 14  ***
## 15
```

```
Sum = as.data.frame(m1)

cldList(p.value ~ contrast, data=Sum)   ### alfa level = 0.05
```

```
##          Group Letter MonoLetter
## 1   Inside,Dsp      a        a
## 2  Inside,Dsuz      b         b
## 3  Outside,Dsp      b         b
## 4 Outside,Dsuz      c          c
## 5     Soil,Dsp      d           d
## 6    Soil,Dsuz      d           d
```

```
m2 <- art.con(m, "hab", adjust="holm") %>%
  summary() %>%  # add significance stars to the output
  mutate(sig. = symnum(p.value, corr=FALSE, na=FALSE,
                     cutpoints = c(0, .001, .01, .05, .10, 1),
                     symbols = c("***", "**", "*", ".", " ")))
```

Habitat Contrast

```
## NOTE: Results may be misleading due to involvement in interactions
```

```
m2
```

```
##           contrast estimate       SE  df  t.ratio       p.value sig.
## 1 Inside - Outside 161.3083 11.44667 714 14.09216  5.700952e-40  ***
## 2    Inside - Soil 383.5792 11.44667 714 33.51011 3.531643e-148  ***
## 3   Outside - Soil 222.2708 11.44667 714 19.41795  1.837929e-67  ***
```

```
Sum = as.data.frame(m2)

cldList(p.value ~ contrast, data=Sum)   ### alfa level = 0.05
```

```
##     Group Letter MonoLetter
## 1  Inside      a        a
## 2 Outside      b         b
## 3    Soil      c          c
```

Fly species contrast

```
m3 <- art.con(m, "fly_sp", adjust="holm") %>%
  summary() %>%  # add significance stars to the output
  mutate(sig. = symnum(p.value, corr=FALSE, na=FALSE,
                     cutpoints = c(0, .001, .01, .05, .10, 1),
                     symbols = c("***", "**", "*", ".", " ")))
```

```
## NOTE: Results may be misleading due to involvement in interactions
```

```
m3
```

```
##     contrast estimate       SE  df  t.ratio      p.value sig.
## 1 Dsp - Dsuz 241.3111 12.22589 714 19.73771 1.547538e-69  ***
```

```
Sum = as.data.frame(m3)

library(rcompanion)

cldList(p.value ~ contrast, data=Sum)   ### alfa level = 0.05
```

```
##   Group Letter MonoLetter
## 1   Dsp      a          a
## 2  Dsuz      b          b
```

Graphic of *T. anastrephae* success with interactions.

```
ggplot(dt, aes(x = factor(hab), y = tric_suc, fill = fly_sp, colour = fly_sp)) +
  geom_violin(trim = T, position=position_dodge(1), scale = "width") +
  scale_y_continuous(breaks=seq(0, 13, 2)) +
  labs(x = "Habitat",
       y = italic('Trichopria anastrephae')~ "success in peach") +
  annotate('text', label='a',
           x=0.77, y= 13.5, hjust=0.1, vjust=0, size=4, color='black') +
  annotate('text', label='b',
           x=1.22, y= 3.5, hjust=0.1, vjust=0, size=4, color='black') +
  annotate('text', label='b',
           x=1.77, y= 7.5, hjust=0.1, vjust=0, size=4, color='black') +
  annotate('text', label='c',
           x=2.22, y= 4.5, hjust=0.1, vjust=0, size=4, color='black') +
  annotate('text', label='d',
           x=2.77, y= 1.5, hjust=0.1, vjust=0, size=4, color='black') +
  annotate('text', label='d',
           x=3.20, y= 1.5, hjust=0.1, vjust=0, size=4, color='black') +
  geom_boxplot(width=0.1, color="black", alpha=0.2, position=position_dodge(1)) +
  geom_vline(xintercept = 1.5) +
  geom_vline(xintercept = 2.5)
```

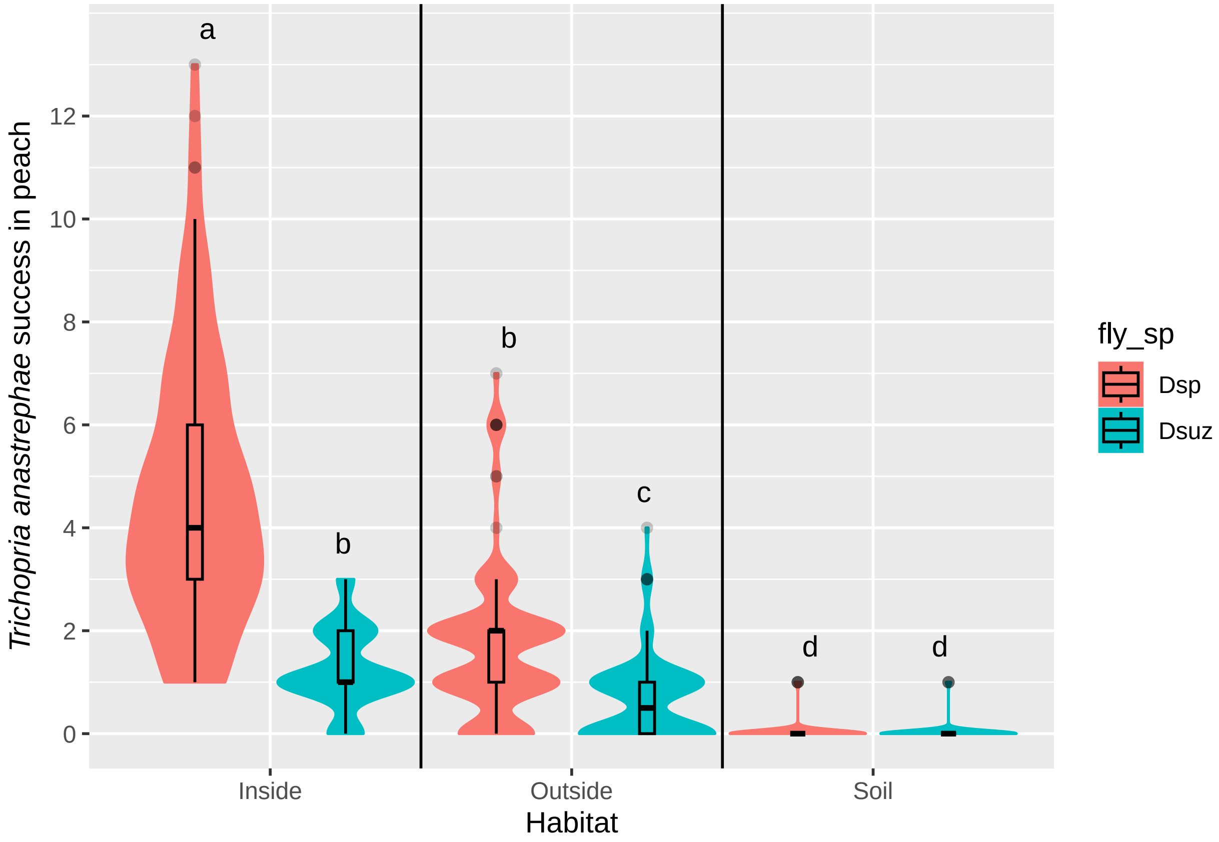

P. vindemiae analysis

Model, sums of aligned responses and ANOVAs on aligned responses not of interest (should all be ~0)

```
m4 <- art(pach_suc ~ hab + fly_sp + hab * fly_sp, data=dt)
summary(m4)
```

```
## Aligned Rank Transform of Factorial Model
##
## Call:
## art(formula = pach_suc ~ hab + fly_sp + hab * fly_sp, data = dt)
##
## Column sums of aligned responses (should all be ~0):
##        hab     fly_sp hab:fly_sp
##          0          0          0
##
## F values of ANOVAs on aligned responses not of interest (should all be ~0):
##    Min. 1st Qu.  Median    Mean 3rd Qu.    Max.
##       0       0       0       0       0       0
```

```
anova(m4, response="aligned")
```

```
## Analysis of Variance of Aligned Rank Transformed Data
##
```

```
## Table Type: Anova Table (Type III tests)
## Model: No Repeated Measures (lm)
## Response: aligned(pach_suc)
##
##              Aligned By Df Df.res F value Pr(>F)
## 1 fly_sp            hab  1    714       0      1
## 2 hab:fly_sp        hab  2    714       0      1
## 3 hab            fly_sp  2    714       0      1
## 4 hab:fly_sp     fly_sp  2    714       0      1
## 5 hab        hab:fly_sp  2    714       0      1
## 6 fly_sp     hab:fly_sp  1    714       0      1
## ---
## Signif. codes:   0 '***' 0.001 '**' 0.01 '*' 0.05 '.' 0.1 ' ' 1
```

```
anova(m4)
```

```
## Analysis of Variance of Aligned Rank Transformed Data
##
## Table Type: Anova Table (Type III tests)
## Model: No Repeated Measures (lm)
## Response: art(pach_suc)
##
##              Df Df.res F value     Pr(>F)
## 1 hab         2    714  85.728 < 2.22e-16 ***
## 2 fly_sp      1    714  84.386 < 2.22e-16 ***
## 3 hab:fly_sp  2    714 113.087 < 2.22e-16 ***
## ---
## Signif. codes:   0 '***' 0.001 '**' 0.01 '*' 0.05 '.' 0.1 ' ' 1
```

Contrasts

```
m5 <- art.con(m4, "hab:fly_sp", adjust="holm") %>%
  summary() %>%  # add significance stars to the output
  mutate(sig. = symnum(p.value, corr=FALSE, na=FALSE,
                       cutpoints = c(0, .001, .01, .05, .10, 1),
                       symbols = c("***", "**", "*", ".", " ")))
m5
```

Interaction contrast

```
##                      contrast   estimate      SE  df     t.ratio      p.value
## 1    Inside,Dsp - Inside,Dsuz   68.66250 20.3062 714   3.3813570 3.802858e-03
## 2    Inside,Dsp - Outside,Dsp -159.80417 20.3062 714  -7.8697241 1.055241e-13
## 3   Inside,Dsp - Outside,Dsuz -149.49583 20.3062 714  -7.3620794 3.499553e-12
## 4       Inside,Dsp - Soil,Dsp   79.07917 20.3062 714   3.8943367 6.462476e-04
## 5      Inside,Dsp - Soil,Dsuz -172.06667 20.3062 714  -8.4736038 1.220157e-15
## 6   Inside,Dsuz - Outside,Dsp -228.46667 20.3062 714 -11.2510811 4.303516e-26
## 7  Inside,Dsuz - Outside,Dsuz -218.15833 20.3062 714 -10.7434364 4.645217e-24
## 8      Inside,Dsuz - Soil,Dsp   10.41667 20.3062 714   0.5129797 1.000000e+00
## 9     Inside,Dsuz - Soil,Dsuz -240.72917 20.3062 714 -11.8549608 1.418348e-28
## 10 Outside,Dsp - Outside,Dsuz   10.30833 20.3062 714   0.5076447 1.000000e+00
## 11      Outside,Dsp - Soil,Dsp  238.88333 20.3062 714  11.7640608 3.253668e-28
## 12     Outside,Dsp - Soil,Dsuz  -12.26250 20.3062 714  -0.6038797 1.000000e+00
## 13     Outside,Dsuz - Soil,Dsp  228.57500 20.3062 714  11.2564161 4.303516e-26
## 14   Outside,Dsuz - Soil,Dsuz  -22.57083 20.3062 714  -1.1115244 1.000000e+00
```

```
## 15       Soil,Dsp - Soil,Dsuz -251.14583 20.3062 714 -12.3679405 8.481637e-31
##    sig.
## 1    **
## 2   ***
## 3   ***
## 4   ***
## 5   ***
## 6   ***
## 7   ***
## 8
## 9   ***
## 10
## 11  ***
## 12
## 13  ***
## 14
## 15  ***
```

```
Sum = as.data.frame(m5)
cldList(p.value ~ contrast, data=Sum)  ### alfa level = 0.05
```

```
##          Group Letter MonoLetter
## 1   Inside,Dsp      a         a
## 2  Inside,Dsuz      b          b
## 3  Outside,Dsp      c           c
## 4 Outside,Dsuz      c           c
## 5     Soil,Dsp      b          b
## 6    Soil,Dsuz      c           c
```

```
m6 <- art.con(m4, "hab", adjust="holm") %>%
  summary() %>%  # add significance stars to the output
  mutate(sig. = symnum(p.value, corr=FALSE, na=FALSE,
                       cutpoints = c(0, .001, .01, .05, .10, 1),
                       symbols = c("***", "**", "*", ".", " ")))
```

Habitat Contrast

```
## NOTE: Results may be misleading due to involvement in interactions
```

```
m6
```

```
##           contrast  estimate       SE  df    t.ratio      p.value sig.
## 1 Inside - Outside -219.4875 16.83026 714 -13.041243 1.518095e-34  ***
## 2    Inside - Soil -126.8750 16.83026 714  -7.538505 2.891193e-13  ***
## 3   Outside - Soil   92.6125 16.83026 714   5.502737 5.215604e-08  ***
```

```
Sum = as.data.frame(m6)
cldList(p.value ~ contrast, data=Sum)  ### alfa level = 0.05
```

```
##     Group Letter MonoLetter
## 1  Inside      a         a
## 2 Outside      b          b
## 3    Soil      c           c
```

Fly species contrast

```
m7 <- art.con(m4, "fly_sp", adjust="holm") %>%
  summary() %>%  # add significance stars to the output
  mutate(sig. = symnum(p.value, corr=FALSE, na=FALSE,
                      cutpoints = c(0, .001, .01, .05, .10, 1),
                      symbols = c("***", "**", "*", ".", " ")))
```

```
## NOTE: Results may be misleading due to involvement in interactions
```

```
m7
```

```
##     contrast  estimate       SE  df   t.ratio      p.value sig.
## 1 Dsp - Dsuz -134.1556 14.60406 714 -9.186183 4.346913e-19  ***
```

```
Sum = as.data.frame(m7)
cldList(p.value ~ contrast, data=Sum)   ### alfa level = 0.05
```

```
##   Group Letter MonoLetter
## 1   Dsp      a          a
## 2  Dsuz      b          b
```

Graphic of *P. vindemmiae* success with interactions

```
ggplot(dt, aes(x = factor(hab), y = pach_suc, fill = fly_sp, colour = fly_sp)) +
  geom_violin(trim = T, position=position_dodge(1), scale = "width") +
  scale_y_continuous(breaks=seq(0, 10, 2)) +
  labs(x = "Habitat",
       y = italic('Pachycrepoideus vindemmiae')~ "success in peach") +
  annotate('text', label='b',
           x=0.75, y=3.5, hjust=0.1, vjust=0, size=4, color='black') +
  annotate('text', label='c',
           x=1.20, y=2.5, hjust=0.1, vjust=0, size=4, color='black') +
  annotate('text', label='a',
           x=1.75, y= 10.5, hjust=0.1, vjust=0, size=4, color='black') +
  annotate('text', label='a',
           x=2.20, y= 6.5, hjust=0.1, vjust=0, size=4, color='black') +
  annotate('text', label='c',
           x=2.75, y= 1.5, hjust=0.1, vjust=0, size=4, color='black') +
  annotate('text', label='a',
           x=3.20, y= 4.5, hjust=0.1, vjust=0, size=4, color='black') +
  geom_boxplot(width=0.1, color="black", alpha=0.2, position=position_dodge(1)) +
  geom_vline(xintercept = 1.5) +
  geom_vline(xintercept = 2.5)
```

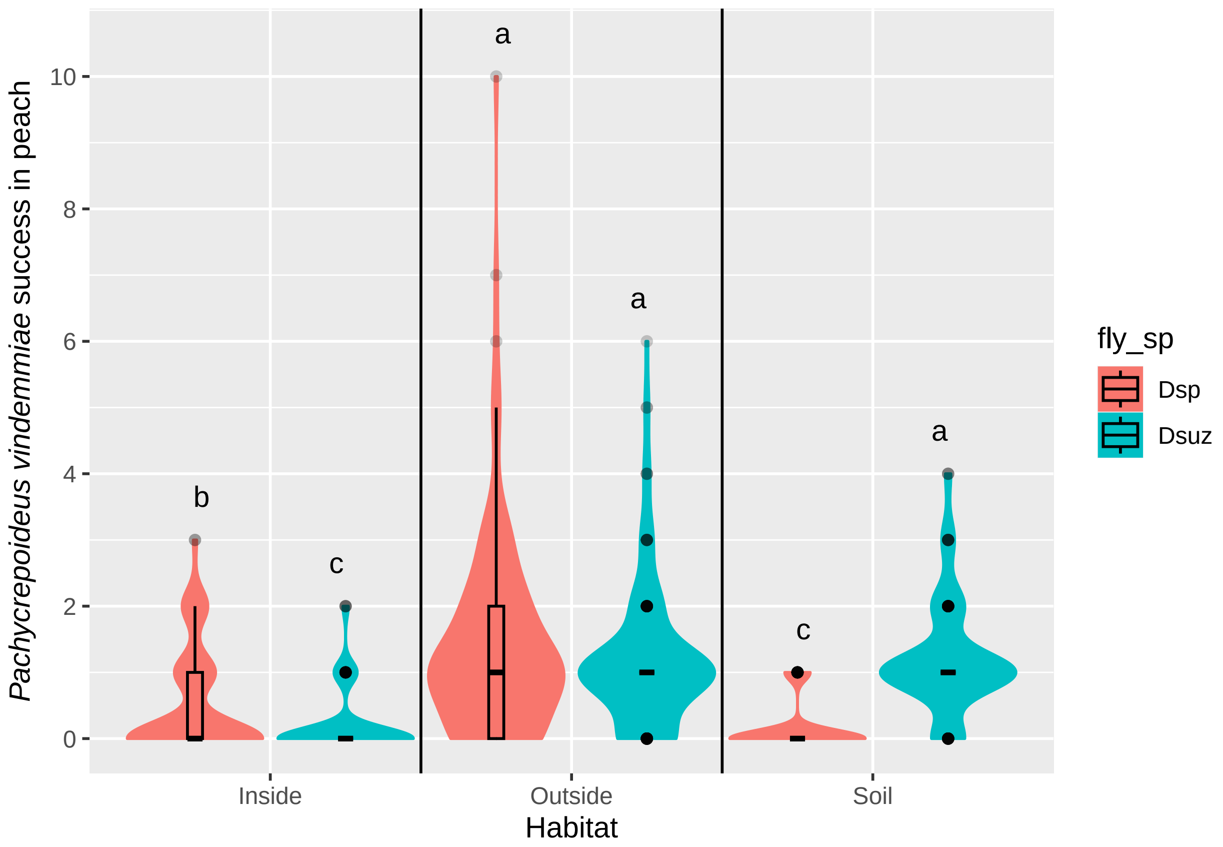

Elkin, Lisa A., Matthew Kay, James J. Higgins, and Jacob O. Wobbrock. 2021. "An Aligned Rank Transform Procedure for Multifactor Contrast Tests." *The 34th Annual ACM Symposium on User Interface Software and Technology*, October. https://doi.org/10.1145/3472749.3474784.

Wobbrock, Jacob O., Leah Findlater, Darren Gergle, and James J. Higgins. 2011. "The Aligned Rank Transform for Nonparametric Factorial Analyses Using Only Anova Procedures." *Proceedings of the SIGCHI Conference on Human Factors in Computing Systems*, May. https://doi.org/10.1145/1978942.1978963.
