# Supplementary material for: Implications of the Niche Partitioning and Coexistence of Two Resident Parasitoids for Drosophila suzukii Management in Non-Crop Areas
Source: Insects. 2023 Feb 23;14(3):222. doi: 10.3390/insects14030222 (PMC10051101; doi:10.3390/insects14030222)
Supplement: Supplementary file 1 [file insects-14-00222-s001.zip › File S4 Statistical Analysis_Habitat-use-by-Drosophilids.pdf]

Habitat use by Drosophilid species

Segundo Núñez Campero

2023-01-29

```
library(readxl)
library(agricolae)
library(ggplot2)

dt <- read_excel("Para hablar con Sergio.xlsx",
                                     sheet = "Planilla_R-Binomial-peach")
View(dt)

dt$hab <- as.factor(dt$hab)
dt$fly_sp <- as.factor(dt$fly_sp)
dt$avb_fly_pu <- as.numeric(dt$avb_fly_pu)
```

Difference in use between drosophilid species in each habitat in peach

```
ins_fly <- subset(dt, hab=="Inside")
out_fly <- subset(dt, hab=="Outside")
soi_fly <- subset(dt, hab=="Soil")

kruskal(ins_fly$avb_fly_pu, ins_fly$fly_sp, alpha = 0.05, p.adj = "holm", console = T)
```

```
##
## Study: ins_fly$avb_fly_pu ~ ins_fly$fly_sp
## Kruskal-Wallis test's
## Ties or no Ties
##
## Critical Value: 166.9431
## Degrees of freedom: 1
## Pvalue Chisq  : 0
##
## ins_fly$fly_sp,  means of the ranks
##
##      ins_fly.avb_fly_pu   r
## Dsp             178.275 120
## Dsuz             62.725 120
##
## Post Hoc Analysis
##
## P value adjustment method: holm
## t-Student: 1.969982
## Alpha    : 0.05
## Minimum Significant Difference: 9.693872
##
## Treatments with the same letter are not significantly different.
```

```
##
##      ins_fly$avb_fly_pu groups
## Dsp             178.275      a
## Dsuz             62.725      b
```

```r
kruskal(out_fly$avb_fly_pu, out_fly$fly_sp, alpha = 0.05, p.adj = "holm", console = T)
```

```
##
## Study: out_fly$avb_fly_pu ~ out_fly$fly_sp
## Kruskal-Wallis test's
## Ties or no Ties
##
## Critical Value: 66.78757
## Degrees of freedom: 1
## Pvalue Chisq  : 3.330669e-16
##
## out_fly$fly_sp,  means of the ranks
##
##      out_fly.avb_fly_pu   r
## Dsp             156.925 120
## Dsuz             84.075 120
##
## Post Hoc Analysis
##
## P value adjustment method: holm
## t-Student: 1.969982
## Alpha    : 0.05
## Minimum Significant Difference: 14.93783
##
## Treatments with the same letter are not significantly different.
##
##      out_fly$avb_fly_pu groups
## Dsp             156.925      a
## Dsuz             84.075      b
```

```r
kruskal(soi_fly$avb_fly_pu, soi_fly$fly_sp, alpha = 0.05, p.adj = "holm", console = T)
```

```
##
## Study: soi_fly$avb_fly_pu ~ soi_fly$fly_sp
## Kruskal-Wallis test's
## Ties or no Ties
##
## Critical Value: 122.7806
## Degrees of freedom: 1
## Pvalue Chisq  : 0
##
## soi_fly$fly_sp,  means of the ranks
##
##      soi_fly.avb_fly_pu   r
## Dsp            72.58333 120
## Dsuz          168.41667 120
##
## Post Hoc Analysis
##
## P value adjustment method: holm
```

```
## t-Student: 1.969982
## Alpha    : 0.05
## Minimum Significant Difference: 11.90597
##
## Treatments with the same letter are not significantly different.
##
##      soi_fly$avb_fly_pu groups
## Dsuz          168.41667      a
## Dsp            72.58333      b
```

Habitat use by Drosophilid species in Peach

```
sub_Dsp <- subset(dt, fly_sp == "Dsp")
sub_Dsuz <- subset(dt, fly_sp == "Dsuz")
View(sub_Dsp)

kruskal(sub_Dsp$avb_fly_pu, alpha= 0.05, sub_Dsp$hab, p.adj = ("holm"), console = T)
```

```
##
## Study: sub_Dsp$avb_fly_pu ~ sub_Dsp$hab
## Kruskal-Wallis test's
## Ties or no Ties
##
## Critical Value: 295.6566
## Degrees of freedom: 2
## Pvalue Chisq  : 0
##
## sub_Dsp$hab,  means of the ranks
##
##         sub_Dsp.avb_fly_pu   r
## Inside           290.18750 120
## Outside          190.44583 120
## Soil              60.86667 120
##
## Post Hoc Analysis
##
## P value adjustment method: holm
## t-Student: 2.405315
## Alpha    : 0.05
## Minimum Significant Difference: 13.55065
##
## Treatments with the same letter are not significantly different.
##
##         sub_Dsp$avb_fly_pu groups
## Inside           290.18750      a
## Outside          190.44583      b
## Soil              60.86667      c
```

```
kruskal(sub_Dsuz$avb_fly_pu, alpha= 0.05, sub_Dsuz$hab, p.adj = ("holm"), console = T)
```

```
##
## Study: sub_Dsuz$avb_fly_pu ~ sub_Dsuz$hab
## Kruskal-Wallis test's
## Ties or no Ties
##
## Critical Value: 40.5116
```

```
## Degrees of freedom: 2
## Pvalue Chisq  : 1.595944e-09
##
## sub_Dsuz$hab,  means of the ranks
##
##          sub_Dsuz.avb_fly_pu   r
## Inside              215.9208 120
## Outside             191.6417 120
## Soil                133.9375 120
##
## Post Hoc Analysis
##
## P value adjustment method: holm
## t-Student: 2.405315
## Alpha    : 0.05
## Minimum Significant Difference: 30.06289
##
## Treatments with the same letter are not significantly different.
##
##          sub_Dsuz$avb_fly_pu groups
## Inside              215.9208      a
## Outside             191.6417      a
## Soil                133.9375      b
```

```
ggplot(dt, aes(x = factor(hab), y = avb_fly_pu, fill = fly_sp)) +
  geom_violin(trim = T, position=position_dodge(1), scale = "width") +
  scale_y_continuous(breaks=seq(0, 30, 2)) +
  labs(x = "Habitat",
       y = "Fly pupae number") + #Título de nuestros ejes
  annotate('text', label='a',
          x= 0.74, y= 29.5, hjust=0.1, vjust=0, size=4, color='black') +
   annotate('text', label='A',
          x= 0.74, y= 4, hjust=0.1, vjust=0, size=4, color='black') +
    annotate('text', label='b',
          x= 1.74, y= 20.5, hjust=0.1, vjust=0, size=4, color='black') +
   annotate('text', label='A',
          x= 1.74, y= -1, hjust=0.1, vjust=0, size=4, color='black') +
    annotate('text', label='c',
          x= 2.74, y= 4.5, hjust=0.1, vjust=0, size=4, color='black') +
   annotate('text', label='A',
          x= 2.74, y= -1, hjust=0.1, vjust=0, size=4, color='black') +
    annotate('text', label= substitute(paste(italic("a"))),
          x= 1.24, y= 11.5, hjust=0.1, vjust=0, size=4, color='black') +
   annotate('text', label='B',
          x= 1.24, y= -1, hjust=0.1, vjust=0, size=4, color='black') +
    annotate('text', label= substitute(paste(italic("a"))),
          x= 2.24, y= 13.5, hjust=0.1, vjust=0, size=4, color='black') +
   annotate('text', label='B',
          x= 2.24, y= 0, hjust=0.1, vjust=0, size=4, color='black') +
   annotate('text', label= substitute(paste(italic("b"))),
          x= 3.24, y= 10.5, hjust=0.1, vjust=0, size=4, color='black') +
   annotate('text', label='B',
          x= 3.24, y= -1, hjust=0.1, vjust=0, size=4, color='black') +
  geom_boxplot(width=0.1, color="black", alpha=0.2, position=position_dodge(1)) +
```

```
  geom_vline(xintercept = 1.5) +
geom_vline(xintercept = 2.5)
```

```
## Warning in is.na(x): is.na() aplicado a un objeto que no es (lista o vector) de
## tipo 'language

## Warning in is.na(x): is.na() aplicado a un objeto que no es (lista o vector) de
## tipo 'language

## Warning in is.na(x): is.na() aplicado a un objeto que no es (lista o vector) de
## tipo 'language
```

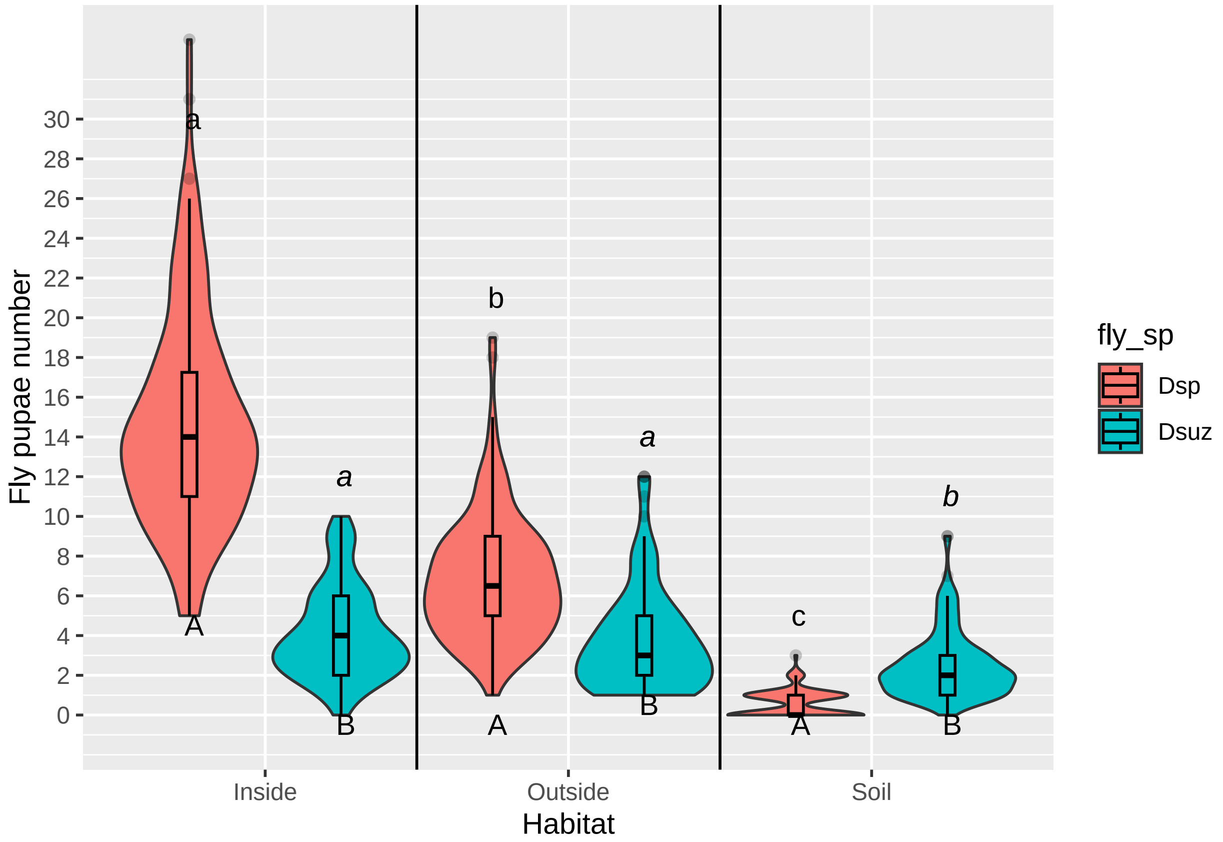

Difference in use between drosophilid species in each habitat in guava

```
library(readxl)
library(agricolae)

dt2 <- read_excel("guava_data.xlsx",
                  sheet = "Hoja1")
View(dt2)

dt2$hab <- as.factor(dt2$hab)
dt2$fly_sp <- as.factor(dt2$fly_sp)
dt2$tric_suc <- as.numeric(dt2$tric_suc)
dt2$pach_suc <- as.numeric(dt2$pach_suc)

attach(dt2)
```

```r
ins_fly <- subset(dt2, hab=="Inside")
out_fly <- subset(dt2, hab=="Outside")
soi_fly <- subset(dt2, hab=="Soil")

kruskal(ins_fly$avb_fly_pu, ins_fly$fly_sp, alpha = 0.05, p.adj = "holm", console = T)
```

```
##
## Study: ins_fly$avb_fly_pu ~ ins_fly$fly_sp
## Kruskal-Wallis test's
## Ties or no Ties
##
## Critical Value: 173.9573
## Degrees of freedom: 1
## Pvalue Chisq  : 0
##
## ins_fly$fly_sp,  means of the ranks
##
##      ins_fly.avb_fly_pu   r
## Dsp           178.99583 120
## Dsuz           62.00417 120
##
## Post Hoc Analysis
##
## P value adjustment method: holm
## t-Student: 1.969982
## Alpha    : 0.05
## Minimum Significant Difference: 9.134957
##
## Treatments with the same letter are not significantly different.
##
##      ins_fly$avb_fly_pu groups
## Dsp           178.99583      a
## Dsuz           62.00417      b
```

```r
kruskal(out_fly$avb_fly_pu, out_fly$fly_sp, alpha = 0.05, p.adj = "holm", console = T)
```

```
##
## Study: out_fly$avb_fly_pu ~ out_fly$fly_sp
## Kruskal-Wallis test's
## Ties or no Ties
##
## Critical Value: 108.9104
## Degrees of freedom: 1
## Pvalue Chisq  : 0
##
## out_fly$fly_sp,  means of the ranks
##
##      out_fly.avb_fly_pu   r
## Dsp                166.7 120
## Dsuz                74.3 120
##
## Post Hoc Analysis
##
```

```
## P value adjustment method: holm
## t-Student: 1.969982
## Alpha    : 0.05
## Minimum Significant Difference: 12.89532
##
## Treatments with the same letter are not significantly different.
##
##      out_fly$avb_fly_pu groups
## Dsp               166.7      a
## Dsuz               74.3      b
```

```
kruskal(soi_fly$avb_fly_pu, soi_fly$fly_sp, alpha = 0.05, p.adj = "holm", console = T)
```

```
##
## Study: soi_fly$avb_fly_pu ~ soi_fly$fly_sp
## Kruskal-Wallis test's
## Ties or no Ties
##
## Critical Value: 4.912938
## Degrees of freedom: 1
## Pvalue Chisq  : 0.02665627
##
## soi_fly$fly_sp,  means of the ranks
##
##      soi_fly.avb_fly_pu   r
## Dsp            111.7792 120
## Dsuz           129.2208 120
##
## Post Hoc Analysis
##
## P value adjustment method: holm
## t-Student: 1.969982
## Alpha    : 0.05
## Minimum Significant Difference: 15.37375
##
## Treatments with the same letter are not significantly different.
##
##      soi_fly$avb_fly_pu groups
## Dsuz           129.2208      a
## Dsp            111.7792      b
```

Habitat use by Drosophilid species in Peach

```
sub_Dsp <- subset(dt2, fly_sp == "Dsp")
sub_Dsuz <- subset(dt2, fly_sp == "Dsuz")

kruskal(sub_Dsp$avb_fly_pu, alpha= 0.05, sub_Dsp$hab, p.adj = ("holm"), console = T)
```

```
##
## Study: sub_Dsp$avb_fly_pu ~ sub_Dsp$hab
## Kruskal-Wallis test's
## Ties or no Ties
##
## Critical Value: 295.6091
## Degrees of freedom: 2
## Pvalue Chisq  : 0
```

```
##
## sub_Dsp$hab,  means of the ranks
##
##          sub_Dsp.avb_fly_pu   r
## Inside           290.10000 120
## Outside          190.28333 120
## Soil              61.11667 120
##
## Post Hoc Analysis
##
## P value adjustment method: holm
## t-Student: 2.405315
## Alpha    : 0.05
## Minimum Significant Difference: 13.53574
##
## Treatments with the same letter are not significantly different.
##
##          sub_Dsp$avb_fly_pu groups
## Inside           290.10000      a
## Outside          190.28333      b
## Soil              61.11667      c
```

```
kruskal(sub_Dsuz$avb_fly_pu, alpha= 0.05, sub_Dsuz$hab, p.adj = ("holm"), console = T)
```

```
##
## Study: sub_Dsuz$avb_fly_pu ~ sub_Dsuz$hab
## Kruskal-Wallis test's
## Ties or no Ties
##
## Critical Value: 5.114484
## Degrees of freedom: 2
## Pvalue Chisq  : 0.07751823
##
## sub_Dsuz$hab,  means of the ranks
##
##          sub_Dsuz.avb_fly_pu   r
## Inside               190.575 120
## Outside              186.275 120
## Soil                 164.650 120
##
## Post Hoc Analysis
##
## P value adjustment method: holm
## t-Student: 2.405315
## Alpha    : 0.05
## Minimum Significant Difference: 29.42533
##
## Treatments with the same letter are not significantly different.
##
##          sub_Dsuz$avb_fly_pu groups
## Inside               190.575      a
## Outside              186.275      a
## Soil                 164.650      a
```

```r
library(ggplot2)

ggplot(dt2, aes(x = factor(hab), y = avb_fly_pu, fill = fly_sp)) +
  geom_violin(trim = T, position=position_dodge(1), scale = "width") +
  scale_y_continuous(breaks=seq(0, 33, 2)) +
  labs(x = "Habitat",
       y = "Fly pupae number in guavas") + #Título de nuestros ejes
  annotate('text', label='a',
           x= 0.74, y= 32.5, hjust=0.1, vjust=0, size=4, color='black') +
  annotate('text', label='A',
           x= 0.74, y= 3.5, hjust=0.1, vjust=0, size=4, color='black') +

  annotate('text', label='b',
           x= 1.74, y= 18.5, hjust=0.1, vjust=0, size=4, color='black') +
   annotate('text', label='A',
           x= 1.74, y= 0, hjust=0.1, vjust=0, size=4, color='black') +
    annotate('text', label='c',
           x= 2.74, y= 5.5, hjust=0.1, vjust=0, size=4, color='black') +
   annotate('text', label='A',
           x= 2.74, y= -2, hjust=0.1, vjust=0, size=4, color='black') +
    annotate('text', label= substitute(paste(italic("a"))),
           x= 1.24, y= 12.5, hjust=0.1, vjust=0, size=4, color='black') +
  annotate('text', label= substitute(paste(italic("B"))),
           x= 1.24, y= -2, hjust=0.1, vjust=0, size=4, color='black') +
    annotate('text', label= substitute(paste(italic("a"))),
           x= 2.24, y= 11.5, hjust=0.1, vjust=0, size=4, color='black') +
  annotate('text', label= substitute(paste(italic("B"))),
           x= 2.24, y= -2, hjust=0.1, vjust=0, size=4, color='black') +
   annotate('text', label= substitute(paste(italic("a"))),
           x= 3.24, y= 8.5, hjust=0.1, vjust=0, size=4, color='black') +
  annotate('text', label= substitute(paste(italic("B"))),
           x= 3.24, y= -2, hjust=0.1, vjust=0, size=4, color='black') +
    geom_boxplot(width=0.1, color="black", alpha=0.2, position=position_dodge(1)) +
geom_vline(xintercept = 1.5) +
geom_vline(xintercept = 2.5)
```

```
## Warning in is.na(x): is.na() aplicado a un objeto que no es (lista o vector) de
## tipo 'language

## Warning in is.na(x): is.na() aplicado a un objeto que no es (lista o vector) de
## tipo 'language

## Warning in is.na(x): is.na() aplicado a un objeto que no es (lista o vector) de
## tipo 'language

## Warning in is.na(x): is.na() aplicado a un objeto que no es (lista o vector) de
## tipo 'language

## Warning in is.na(x): is.na() aplicado a un objeto que no es (lista o vector) de
## tipo 'language

## Warning in is.na(x): is.na() aplicado a un objeto que no es (lista o vector) de
## tipo 'language
```

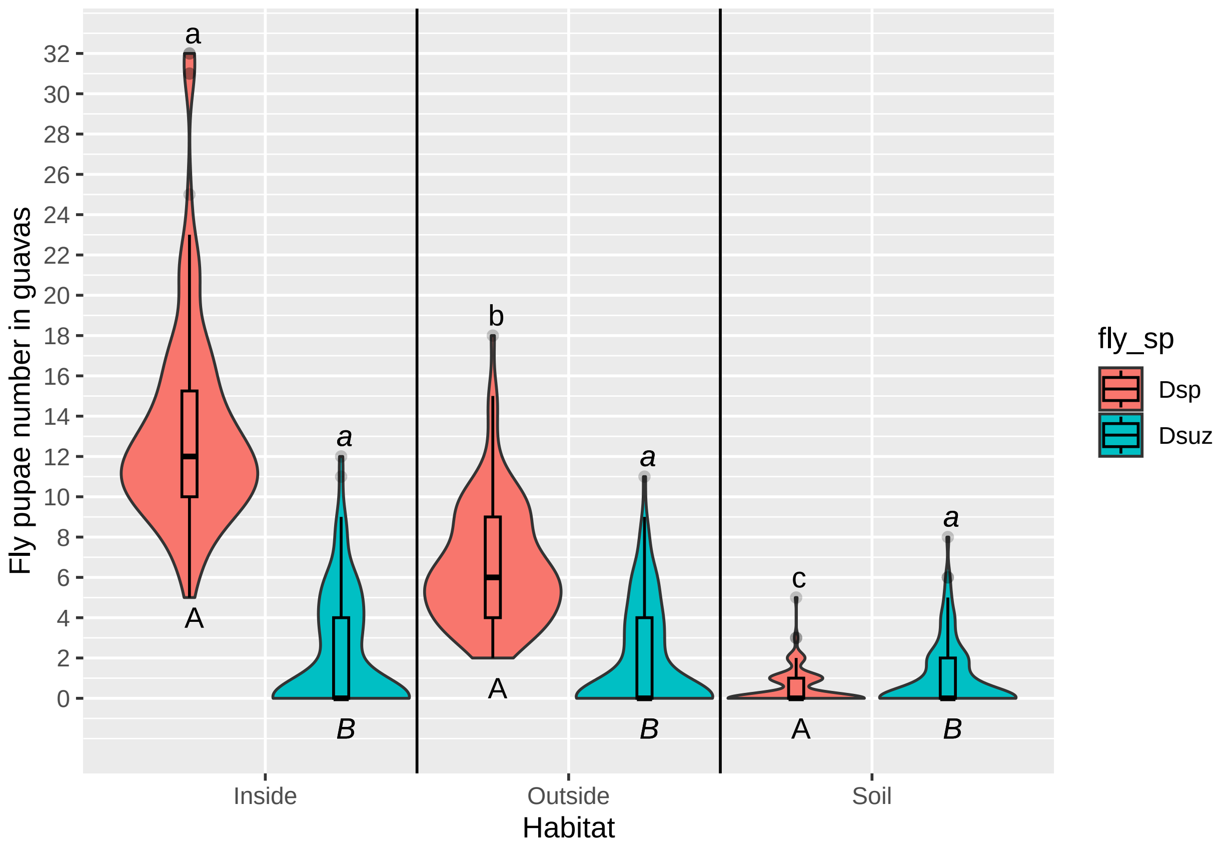
a
b
c
a
a
a
A
A
A
B
B
B
Fly pupae number in guavas
Habitat
Inside
Outside
Soil
fly_sp
Dsp
Dsuz
